# Supplementary material for: Models, components and outcomes of palliative and end-of-life care provided to adults living at home: A systematic umbrella review of reviews
Source: Palliat Med. 2025 Sep 4;39(10):1037–62. doi: 10.1177/02692163251362567 (PMC12640366; doi:10.1177/02692163251362567)
Supplement: sj-docx-1-pmj-10.1177_02692163251362567 – Supplemental material for Models, components and outcomes of palliative and end-of-life care provided to adults living at home: A systematic umbrella review of reviews [file sj-docx-1-pmj-10.1177_02692163251362567.docx]

# **Supplemental material 1**: Completed PRIOR Checklist

Adapted from Gates M, Gates A, Pieper D, et al. Reporting guideline for overviews of reviews of healthcare interventions: development of the PRIOR statement. BMJ 2022;378:e070849. doi:10.1136/bmj-2022-070849.

| **Section**  *Topic* | **#** | **Item** | **Location reported** |
| --- | --- | --- | --- |
| **Title** | | | |
| Title | 1 | Identify the report as an overview of reviews. | p. 1 |
| **Abstract** | | | |
| Abstract | 2 | Provide a comprehensive and accurate summary of the purpose, methods, and results of the overview of reviews. | p. 2 |
| **Introduction** | | | |
| Rationale | 3 | Describe the rationale for conducting the overview of reviews in the context of existing knowledge. | p. 4 |
| Objectives | 4 | Provide an explicit statement of the objective(s) or question(s) addressed by the overview of reviews. | p. 5 |
| **Methods** | | | |
| Eligibility criteria | 5a | Specify the inclusion and exclusion criteria for the overview of reviews. If supplemental primary studies were included, this should be stated, with a rationale. | p. 6 – 7 |
|  | 5b | Specify the definition of ‘systematic review’ as used in the inclusion criteria for the overview of reviews. | p. 7 |
| Information sources | 6 | Specify all databases, registers, websites, organizations, reference lists, and other sources searched or consulted to identify systematic reviews and supplemental primary studies (if included). Specify the date when each source was last searched or consulted. | p. 7 |
| Search strategy | 7 | Present the full search strategies for all databases, registers and websites, such that they could be reproduced. Describe any search filters and limits applied. | Supplemental material 2 and p. 7 |
| Selection process | 8a | Describe the methods used to decide whether a systematic review or supplemental primary study (if included) met the inclusion criteria of the overview of reviews. | p. 8 |
|  | 8b | Describe how overlap in the populations, interventions, comparators, and/or outcomes of systematic reviews was identified and managed during study selection. | Supplemental material 5 and p.8 |
| Data collection process | 9a | Describe the methods used to collect data from reports. | p. 8 |
|  | 9b | If applicable, describe the methods used to identify and manage primary study overlap at the level of the comparison and outcome during data collection. For each outcome, specify the method used to illustrate and/or quantify the degree of primary study overlap across systematic reviews. | Supplemental material 5 and p.8 |
|  | 9c | If applicable, specify the methods used to manage discrepant data across systematic reviews during data collection. | Not applicable |
| Data items | 10 | List and define all variables and outcomes for which data were sought. Describe any assumptions made and/or measures taken to identify and clarify missing or unclear information. | Box 1 and p. 8 |
| Risk of bias | 11a | Describe the methods used to *assess* risk of bias or methodological quality of the included systematic reviews. | p. 9 |
|  | 11b | Describe the methods used to *collect* data on (from the systematic reviews) and/or *assess* the risk of bias of the primary studies included in the systematic reviews. Provide a justification for instances where flawed, incomplete, or missing assessments are identified but not re-assessed. | p. 8, and p. 9 |
|  | 11c | Describe the methods used to *assess* the risk of bias of supplemental primary studies (if included). | Not applicable |
| Synthesis methods | 12a | Describe the methods used to summarize or synthesize results and provide a rationale for the choice(s). | p. 10 |
|  | 12b | Describe any methods used to explore possible causes of heterogeneity among results. | p. 10 |
|  | 12c | Describe any sensitivity analyses conducted to assess the robustness of the synthesized results. | Not applicable |
| Reporting bias assessment | 13 | Describe the methods used to *collect* data on (from the systematic reviews) and/or *assess* the risk of bias due to missing results in a summary or synthesis (arising from reporting biases at the levels of the systematic reviews, primary studies, and supplemental primary studies, if included). | p. 9 |
| Certainty assessment | 14 | Describe the methods used to *collect* data on (from the systematic reviews) and/or *assess* certainty (or confidence) in the body of evidence for an outcome. | p. 9 |
| **Results** | | | |
| Systematic review and supplemental primary study selection | 15a | Describe the results of the search and selection process, including the number of records screened, assessed for eligibility, and included in the overview of reviews, ideally with a flow diagram. | PRISMA p.13 |
|  | 15b | Provide a list of studies that might appear to meet the inclusion criteria, but were excluded, with the main reason for exclusion. | Supplemental material 3 and p. 13 |
| Characteristics of systematic reviews and supplemental primary studies | 16 | Cite each included systematic review and supplemental primary study (if included) and present its characteristics. | Supplemental material 4 and p. 10 |
| Primary study overlap | 17 | Describe the extent of primary study overlap across the included systematic reviews. | Supplemental material 5 and p. 11 |
| Risk of bias in systematic reviews, primary studies, and supplemental primary studies | 18a | Present assessments of risk of bias or methodological quality for each included systematic review. | Supplementary material 4 and p. 12 |
|  | 18b | Present assessments (*collected* from systematic reviews or *assessed* anew) of the risk of bias of the primary studies included in the systematic reviews. | We assessed at review-level, see 18a |
|  | 18c | Present assessments of the risk of bias of supplemental primary studies (if included). | Not applicable |
| Summary or synthesis of results | 19a | For all outcomes, summarize the evidence from the systematic reviews and supplemental primary studies (if included). If meta-analyses were done, present for each the summary estimate and its precision and measures of statistical heterogeneity. If comparing groups, describe the direction of the effect. | p. 14 – 31 |
|  | 19b | If meta-analyses were done, present results of all investigations of possible causes of heterogeneity. | Not applicable |
|  | 19c | If meta-analyses were done, present results of all sensitivity analyses conducted to assess the robustness of synthesized results. | Not applicable |
| Reporting biases | 20 | Present assessments (*collected* from systematic reviews and/or *assessed* anew) of the risk of bias due to missing primary studies, analyses, or results in a summary or synthesis (arising from reporting biases at the levels of the systematic reviews, primary studies, and supplemental primary studies, if included) for each summary or synthesis assessed. | p. 11 and p. 12 |
| Certainty of evidence | 21 | Present assessments (*collected* or *assessed* anew) of certainty (or confidence) in the body of evidence for each outcome. | p. 11 and p. 12 |
| **Discussion** | | | |
| Discussion | 22a | Summarize the main findings, including any discrepancies in findings across the included systematic reviews and supplemental primary studies (if included). | p. 31 - 32 |
|  | 22b | Provide a general interpretation of the results in the context of other evidence. | p. 32 – 33 |
|  | 22c | Discuss any limitations of the evidence from systematic reviews, their primary studies, and supplemental primary studies (if included) included in the overview of reviews. Discuss any limitations of the overview of reviews methods used. | p. 33 |
|  | 22d | Discuss implications for practice, policy, and future research (both systematic reviews and primary research). Consider the relevance of the findings to the end users of the overview of reviews, e.g., healthcare providers, policymakers, patients, among others. | p. 33 – 34 |
| **Other information** | | | |
| Registration and protocol | 23a | Provide registration information for the overview of reviews, including register name and registration number, or state that the overview of reviews was not registered. | p. 5 |
|  | 23b | Indicate where the overview of reviews protocol can be accessed, or state that a protocol was not prepared. | p. 5 |
|  | 23c | Describe and explain any amendments to information provided at registration or in the protocol. Indicate the stage of the overview of reviews at which amendments were made. | p. 34 |
| Support | 24 | Describe sources of financial or non-financial support for the overview of reviews, and the role of the funders or sponsors in the overview of reviews. | p. 34 – 35 |
| Competing interests | 25 | Declare any competing interests of the overview of reviews' authors. | p. 35 |
| Author information | 26a | Provide contact information for the corresponding author. | p. 1 |
|  | 26b | Describe the contributions of individual authors and identify the guarantor of the overview of reviews. | p. 34 |
| Availability of data and other materials | 27 | Report which of the following are available, where they can be found, and under which conditions they may be accessed: template data collection forms; data collected from included systematic reviews and supplemental primary studies; analytic code; any other materials used in the overview of reviews. | p. 35 |

# **Supplemental material 2: Full search strategies for original main database searches**

Structure: (Palliative care AND (primary care OR community/home based care OR OOH) OR (Primary care AND OOH)) AND systematic review filter(s)

Update searches were performed in September 2022, August 2023 and August 2024 by re-running each search and limiting the results to those added to the database since the date of the last search. The final update search was performed on 01 August 2024.

Forward and backwards citation searches were performed using Citation Chaser on all included reviews following each update. The final citation search was performed on 28^th^ August 2024

Ovid MEDLINE(R) ALL <1946 to September 22, 2021>

1 exp advance care planning/ 10224

2 exp attitude to death/ 16526

3 exp bereavement/ 14016

4 death/ 18437

5 hospices/ or "Hospice and Palliative Care Nursing"/ 6643

6 life support care/ 7835

7 palliative care/ or Palliative Medicine/ 58128

8 exp terminal care/ or respite care/ 54815

9 terminally ill/ 6682

10 palliat$.af. 130902

11 hospice$.af. 37411

12 (terminal care or respite care).af. 32298

13 or/1-12 226478

14 journal of palliative care.jn. 1580

15 journal of palliative medicine.jn. 5247

16 hospice journal physical psychosocial & pastoral care of the dying.jn. 348

17 supportive care in cancer.jn. 7696

18 palliative medicine.jn. 2916

19 palliative & supportive care.jn. 1513

20 journal of supportive oncology.jn. 633

21 journal of social work in end of life & palliative care.jn. 404

22 journal of pain & symptom management.jn. 6058

23 journal of pain & palliative care pharmacotherapy.jn. 1068

24 international journal of palliative nursing.jn. 2030

25 death studies.jn. 1688

26 death education.jn. 90

27 american journal of hospice care.jn. 262

28 american journal of hospice & palliative medicine.jn. 3066

29 omega journal of death & dying.jn. 1038

30 or/14-29 35637

31 13 or 30 235928

32 bereave*.mp. 10808

33 attitude to death.mp. 16609

34 end of life.af. 28026

35 Advance* Care.af. 10913

36 ((advanced or terminal*) adj (ill* or disease)).ti,ab,kw. 29655

37 supportive care.ti,ab,kw. 18115

38 dying.ti,ab,kw. 36891

39 "last year of life".ti,ab,kw. 722

40 (limited life adj (expectanc* or span*)).ti,ab,kw. or life-limiting.mp. 3642

41 or/32-40 132628

42 31 or 41 [palliative care concept] 307708

43 exp Primary Health Care/ 174033

44 exp General Practice/ 76472

45 family practi*.af. 88206

46 general pract*.af. or (generalist* or nonspecialist* or non-specialist*).ti,ab,kw. 137182

47 ((primary adj2 care) or (primary adj2 (provider* or setting* or service*))).ti,ab,kw. 151943

48 or/43-47 [primary care concept] 417576

49 exp home care services/ or home health nursing/ or home nursing/ 49050

50 ((home or community) adj5 (care or nursing)).mp. 138956

51 ((hospice or hospital) adj2 home).mp. 5909

52 home-based.ti,ab,kw. 12076

53 exp Community Health Nursing/ or general practitioners/ or physicians, primary care/ or Primary Care Nursing/ or ((primary or practice) adj2 nurs*).ti,ab,kw. or (general practitioner* or GP).ti,ab,kw. 144587

54 Pharmacists/ or (family adj (physician* or doctor*)).ti,ab,kw. or community paramedic*.ti,ab,kw. 38679

55 *community health services/ or community pharmacy services/ or Home Health Aides/ or Community Health Workers/ or Physical Therapists/ or Occupational Therapists/ 34188

56 exp Telemedicine/ or (telemedicine or telehealth).ti,ab,kw. 46044

57 (community adj2 (care or health*)).ti,ab,kw. or community.ti,kw. 187875

58 (district adj nurs*).ti,ab,kw. 1942

59 or/49-58 [home-based or community setting concept] 514250

60 exp After-Hours Care/ 2026

61 Night Care/ 1393

62 (after hour* or ((outside or out or after or off) adj2 (normal or working or office) adj2 (time or hour*))).ti,ab,kw. 2711

63 after office hour*.ti,ab,kw. 48

64 (out of hours or (OOH or OOHs)).ti,ab,kw. 3686

65 out of office hours.ti,ab,kw. 55

66 (off adj hour*).ti,ab,kw. 449

67 ((weekend* or evening* or holiday* or night*) adj (hour* or care*)).ti,ab,kw. 1335

68 ((24 hour* or 24H or around-the-clock or around the clock) adj2 care*).ti,ab,kw. 529

69 or/60-68 [OOh concept] 10480

70 review.pt. 2863516

71 (medline or medlars or embase or pubmed or cochrane).tw,sh. 268028

72 (scisearch or psychinfo or psycinfo).tw,sh. 44844

73 (psychlit or psyclit).tw,sh. 917

74 cinahl.tw,sh. 34009

75 ((hand adj2 search$) or (manual$ adj2 search$)).tw,sh. 14614

76 (electronic database$ or bibliographic database$ or computeri?ed database$ or online database$).tw,sh. 45703

77 (pooling or pooled or mantel haenszel).tw,sh. 122604

78 (peto or dersimonian or der simonian or fixed effect).tw,sh. 8752

79 (retraction of publication or retracted publication).pt. 19974

80 or/71-79 416184

81 70 and 80 177157

82 meta-analysis.pt. 142222

83 meta-analysis.sh. 142222

84 (meta-analys$ or meta analys$ or metaanalys$).tw,sh. 242514

85 (systematic$ adj5 review$).tw,sh. 249920

86 (systematic$ adj5 overview$).tw,sh. 2759

87 (quantitativ$ adj5 review$).tw,sh. 9056

88 (quantitativ$ adj5 overview$).tw,sh. 355

89 (quantitativ$ adj5 synthesis$).tw,sh. 3570

90 (methodologic$ adj5 review$).tw,sh. 7250

91 (methodologic$ adj5 overview$).tw,sh. 482

92 (integrative research review$ or research integration).tw. 155

93 ((qualitativ$ adj5 synthesis$) or (scoping adj review)).tw,sh. 15078

94 Systematic Review.pt. 169097

95 or/82-94 398261

96 81 or 95 [BMJ SR filter] 463828

97 42 and (48 or 59 or 69) [Pall AND primary care or community or OOH] 29815

98 48 and 69 [Primary care and OOH] 2205

99 97 or 98 31873

100 limit 99 to (systematic reviews pre 2019 or systematic reviews) 1638

101 96 and 99 1262

102 100 or 101 1869

## Embase <1974 to 2021 September 22>

1 advance care planning/ 4033

2 attitude to death/ 11271

3 bereavement/ 9666

4 death/ 276755

5 hospice/ 14338

6 exp palliative therapy/ 120991

7 respite care/ 1200

8 terminal care/ or hospice care/ 47165

9 exp terminally ill patient/ 8972

10 palliat$.af. 199702

11 hospice$.af. 57796

12 (terminal care or respite care).af. 40481

13 supportive care.ti,ab,kw. 33536

14 bereave$.mp. 14408

15 attitude to death.mp. 11426

16 end of life.af. 40653

17 ((advanced or terminal* or critical*) adj (ill* or disease)).ti,ab,kw. 132660

18 Advance* Care.af. 12717

19 (limited life adj (expectanc* or span*)).ti,ab,kw. or life-limiting.mp. 5631

20 "last year of life".ti,ab,kw. 1023

21 dying.ti,ab,kw. 47393

22 or/1-21 [palliative concept] 719997

23 exp primary health care/ 183894

24 general practice/ 80137

25 family practi*.af. 25876

26 general pract*.af. or (generalist* or nonspecialist* or non-specialist*).ti,ab,kw. 252972

27 ((primary adj2 care) or (primary adj2 (provider* or setting* or service*))).ti,ab,kw. 204288

28 or/23-27 [primary care concept] 462300

29 exp home care/ 79293

30 ((home or community) adj5 (care or nursing)).mp. 249715

31 ((hospice or hospital) adj2 home).mp. 8843

32 home-based.ti,ab,kw. 16663

33 exp community health nursing/ 23871

34 general practitioner/ or (general practitioner* or GP).ti,ab,kw. 184323

35 ((primary or practice) adj2 nurs*).ti,ab,kw. 35608

36 exp pharmacist/ or paramedical personnel/ 98838

37 (family adj (physician* or doctor*)).ti,ab,kw. 25741

38 community care/ or community based rehabilitation/ or community health nursing/ or community integration/ or community mental health service/ or community program/ or preventive health service/ 111275

39 "pharmacy (shop)"/ 6435

40 health auxiliary/ 7815

41 physiotherapist/ 23183

42 occupational therapist/ 7340

43 community paramedic*.ti,ab,kw. 200

44 telehealth/ or telemedicine/ or telenursing/ 43158

45 (telemedicine or telehealth or telenursing).ti,ab,kw. 31193

46 (community adj2 (care or health*)).ti,ab,kw. or community.ti,kw. 223520

47 (district adj nurs*).ti,ab,kw. 1886

48 or/29-47 [home-based or community setting concept] 838128

49 out-of-hours care/ 526

50 night care/ 189

51 (after hour* or ((outside or out or after or off) adj2 (normal or working or office) adj2 (time or hour*))).ti,ab,kw. 4075

52 after office hour*.ti,ab,kw. 79

53 (out of hours or (OOH or OOHs)).ti,ab,kw. 5348

54 out of office hours.ti,ab,kw. 78

55 (off adj hour*).ti,ab,kw. 805

56 ((weekend* or evening* or holiday* or night*) adj (hour* or care*)).ti,ab,kw. 1864

57 ((24 hour* or 24H or around-the-clock or around the clock) adj2 care*).ti,ab,kw. 820

58 or/49-57 [OOH concept] 12737

59 exp review/ 2829076

60 (literature adj3 review$).ti,ab. 389148

61 exp meta analysis/ 226698

62 exp "systematic review"/ 314005

63 or/59-62 3140183

64 (medline or medlars or embase or pubmed or cinahl or amed or psychlit or psyclit or psychinfo or psycinfo or scisearch or cochrane).ti,ab. 350843

65 RETRACTED ARTICLE/ 11162

66 64 or 65 361629

67 63 and 66 278429

68 ((systematic$ or scoping) adj2 (review$ or overview)).ti,ab. 299200

69 (meta?anal$ or meta anal$ or meta-anal$ or metaanal$ or metanal$).ti,ab. 276350

70 67 or 68 or 69 532221

71 22 and (28 or 48 or 58) [Pall AND (primary care or community or OOH)] 54661

72 28 and 58 [primary care AND OOH] 2240

73 71 or 72 56693

74 73 and 70 [limited to SRs] 1884

75 limit 74 to conference abstract status 518

76 74 not 75 1366

The Cochrane Library

Comment: Search for palliative care AND (community health, OOH, primary care).........and also (primary care AND OOH). No SR filter needed as just results from CDSR used

ID Search

#1 MeSH descriptor: [Advance Care Planning] explode all trees

#2 MeSH descriptor: [Attitude to Death] explode all trees

#3 MeSH descriptor: [Bereavement] explode all trees

#4 MeSH descriptor: [Death] this term only

#5 MeSH descriptor: [Hospices] this term only

#6 MeSH descriptor: [Life Support Care] this term only

#7 MeSH descriptor: [Palliative Care] explode all trees

#8 MeSH descriptor: [Respite Care] explode all trees

#9 MeSH descriptor: [Terminal Care] explode all trees

#10 MeSH descriptor: [Terminally Ill] explode all trees

#11 (palliat* or hospice*):ti,ab,kw

#12 ((terminal or supportive) next care):ti,ab,kw

#13 (respite next care):ti,ab,kw

#14 (bereave* or dying):ti,ab,kw

#15 ("attitude to death"):ti,ab,kw

#16 (((advanced or terminal* or critical*) next (ill* or disease))):ti,ab,kw

#17 (advance* next care):ti,ab,kw

#18 ("last year of life" or (limited next (expectanc* or span*)) or life-limiting):ti,ab,kw

#19 #1 or #2 or #3 or #4 or #5 or #6 or #7 or #8 or #9 or #10 or #11 or #12 or #13 or #14 or #15 or #16 or #17 or #18

#20 MeSH descriptor: [Primary Health Care] explode all trees

#21 MeSH descriptor: [General Practice] explode all trees

#22 (family practi*):ti,ab,kw

#23 (general pract*):ti,ab,kw

#24 (generalist* or nonspecialist* or non-specialist*):ti,ab,kw

#25 (primary near/2 care):ti,ab,kw

#26 (primary near/2 (provider* or setting* or service*)):ti,ab,kw

#27 #20 or #21 or #22 or #23 or #24 or #25 or #26

#28 MeSH descriptor: [Home Care Services] explode all trees

#29 MeSH descriptor: [Home Health Nursing] explode all trees

#30 MeSH descriptor: [Home Nursing] explode all trees

#31 (((home or community) near/5 (care or nursing))):ti,ab,kw

#32 ((hospice or hospital) near/2 home):ti,ab,kw

#33 (home-based):ti,ab,kw

#34 MeSH descriptor: [Community Health Nursing] explode all trees

#35 MeSH descriptor: [General Practitioners] explode all trees

#36 MeSH descriptor: [Physicians, Primary Care] explode all trees

#37 (((district or primary or practice) near/2 nurs*)):ti,ab,kw

#38 MeSH descriptor: [Pharmacists] explode all trees

#39 ((family next (physician* or doctor*))):ti,ab,kw

#40 (general practitioner* or GP):ti,ab,kw

#41 MeSH descriptor: [Community Health Services] this term only

#42 MeSH descriptor: [Community Pharmacy Services] explode all trees

#43 MeSH descriptor: [Home Health Aides] explode all trees

#44 MeSH descriptor: [Community Health Workers] this term only

#45 MeSH descriptor: [Physical Therapists] explode all trees

#46 MeSH descriptor: [Occupational Therapists] explode all trees

#47 MeSH descriptor: [Telemedicine] explode all trees

#48 (telemedicine or telehealth or telenursing):ti,ab,kw

#49 (community near/2 (care or health*)):ti,ab,kw

#50 (community):ti

#51 (community paramedic*):ti,ab,kw

#52 {or #28-#51}

#53 MeSH descriptor: [After-Hours Care] explode all trees

#54 MeSH descriptor: [Night Care] explode all trees

#55 (after next hour*):ti,ab,kw

#56 (((outside or out or after or off) near/2 (normal or working or office) near/2 (time or hour*))):ti,ab,kw

#57 ("after office hour*"):ti,ab,kw

#58 ("(out of hours" or out-of-hours or OOH or OOHs)):ti,ab,kw

#59 ((weekend* or evening* or holiday* or night*) next (hour* or care*))

#60 (((24 next hour* or 24H or around-the-clock or "around the clock") near/2 care*)):ti,ab,kw

#61 {or #53-#60}

#62 #19 and (#27 or #52 or #61)

#63 #27 and #61

#64 #62 or #63

## CINAHL

| **#** | **Query** | **Limiters/Expanders** | **Last Run Via** | **Results** |
| --- | --- | --- | --- | --- |
| S1 | ( (MH "Terminal Care+") or (MH "Palliative Care") or (MH "Attitude to Death") or (MH "Advance Care Planning") or (MH "Respite Care") or (MH "Hospices") or (MH "Life Support Care") ) OR TI ( bereave* or hospice* or "end of life" of "terminally ill" or palliat* ) OR AB ( bereave* or hospice* or "end of life" of "terminally ill" or palliat* ) | Search modes - Boolean/Phrase | Interface - EBSCOhost Research Databases  Search Screen - Advanced Search  Database - CINAHL Complete | Display |
| S2 | TI life-limiting OR AB life-limiting | Search modes - Boolean/Phrase | Interface - EBSCOhost Research Databases  Search Screen - Advanced Search  Database - CINAHL Complete | Display |
| S3 | TI ( ((advanced or terminal* or critical*) n1 (ill* or disease)) ) OR AB ( ((advanced or terminal* or critical*) n1 (ill* or disease)) ) | Search modes - Boolean/Phrase | Interface - EBSCOhost Research Databases  Search Screen - Advanced Search  Database - CINAHL Complete | Display |
| S4 | TI ( (limited life N1 (expectanc* or span*)) ) OR AB ( (limited life N1 (expectanc* or span*)) ) | Expanders - Apply equivalent subjects  Search modes - Boolean/Phrase | Interface - EBSCOhost Research Databases  Search Screen - Advanced Search  Database - CINAHL Complete | Display |
| S5 | S1 OR S2 OR S3 OR S4 | Expanders - Apply equivalent subjects  Search modes - Boolean/Phrase | Interface - EBSCOhost Research Databases  Search Screen - Advanced Search  Database - CINAHL Complete | Display |
| S6 | (MH "Primary Health Care") | Expanders - Apply equivalent subjects  Search modes - Boolean/Phrase | Interface - EBSCOhost Research Databases  Search Screen - Advanced Search  Database - CINAHL Complete | Display |
| S7 | (MH "Family Practice") | Expanders - Apply equivalent subjects  Search modes - Boolean/Phrase | Interface - EBSCOhost Research Databases  Search Screen - Advanced Search  Database - CINAHL Complete | Display |
| S8 | TI family practi* OR AB family practi* | Expanders - Apply equivalent subjects  Search modes - Boolean/Phrase | Interface - EBSCOhost Research Databases  Search Screen - Advanced Search  Database - CINAHL Complete | Display |
| S9 | ( generalist* or nonspecialist* or non-specialist* ) AND ( generalist* or nonspecialist* or non-specialist* ) | Expanders - Apply equivalent subjects  Search modes - Boolean/Phrase | Interface - EBSCOhost Research Databases  Search Screen - Advanced Search  Database - CINAHL Complete | Display |
| S10 | TI primary N2 care OR AB primary N2 care | Expanders - Apply equivalent subjects  Search modes - Boolean/Phrase | Interface - EBSCOhost Research Databases  Search Screen - Advanced Search  Database - CINAHL Complete | Display |
| S11 | TI ( primary N2 (provider* or setting* or service*) ) OR AB ( primary N2 (provider* or setting* or service*) ) | Expanders - Apply equivalent subjects  Search modes - Boolean/Phrase | Interface - EBSCOhost Research Databases  Search Screen - Advanced Search  Database - CINAHL Complete | Display |
| S12 | S6 OR S7 OR S8 OR S9 OR S10 OR S11 | Expanders - Apply equivalent subjects  Search modes - Boolean/Phrase | Interface - EBSCOhost Research Databases  Search Screen - Advanced Search  Database - CINAHL Complete | Display |
| S13 | (MH "Home Health Care+") OR (MH "Home Nursing") | Expanders - Apply equivalent subjects  Search modes - Boolean/Phrase | Interface - EBSCOhost Research Databases  Search Screen - Advanced Search  Database - CINAHL Complete | Display |
| S14 | (MH "Home Health Aides") | Expanders - Apply equivalent subjects  Search modes - Boolean/Phrase | Interface - EBSCOhost Research Databases  Search Screen - Advanced Search  Database - CINAHL Complete | Display |
| S15 | TI ( ((home or community) N5 (care or nursing)) ) OR AB ( ((home or community) N5 (care or nursing)) ) | Expanders - Apply equivalent subjects  Search modes - Boolean/Phrase | Interface - EBSCOhost Research Databases  Search Screen - Advanced Search  Database - CINAHL Complete | Display |
| S16 | TI ( ((hospice or hospital) N2 home) ) OR AB ( ((hospice or hospital) N2 home) ) | Expanders - Apply equivalent subjects  Search modes - Boolean/Phrase | Interface - EBSCOhost Research Databases  Search Screen - Advanced Search  Database - CINAHL Complete | Display |
| S17 | TI home-based OR AB home-based | Expanders - Apply equivalent subjects  Search modes - Boolean/Phrase | Interface - EBSCOhost Research Databases  Search Screen - Advanced Search  Database - CINAHL Complete | Display |
| S18 | (MH "Community Health Nursing") | Expanders - Apply equivalent subjects  Search modes - Boolean/Phrase | Interface - EBSCOhost Research Databases  Search Screen - Advanced Search  Database - CINAHL Complete | Display |
| S19 | (MH "Physicians, Family") | Expanders - Apply equivalent subjects  Search modes - Boolean/Phrase | Interface - EBSCOhost Research Databases  Search Screen - Advanced Search  Database - CINAHL Complete | Display |
| S20 | (MH "Primary Nursing") | Expanders - Apply equivalent subjects  Search modes - Boolean/Phrase | Interface - EBSCOhost Research Databases  Search Screen - Advanced Search  Database - CINAHL Complete | Display |
| S21 | TI ( ((district or primary or practice) N2 nurs*) ) OR AB ( ((district or primary or practice) N2 nurs*) ) | Expanders - Apply equivalent subjects  Search modes - Boolean/Phrase | Interface - EBSCOhost Research Databases  Search Screen - Advanced Search  Database - CINAHL Complete | Display |
| S22 | (MH "Pharmacists") | Expanders - Apply equivalent subjects  Search modes - Boolean/Phrase | Interface - EBSCOhost Research Databases  Search Screen - Advanced Search  Database - CINAHL Complete | Display |
| S23 | TI ( (family n1 (physician* or doctor*)) ) OR AB ( (family n1 (physician* or doctor*)) ) | Expanders - Apply equivalent subjects  Search modes - Boolean/Phrase | Interface - EBSCOhost Research Databases  Search Screen - Advanced Search  Database - CINAHL Complete | Display |
| S24 | (MH "Community Health Services") | Expanders - Apply equivalent subjects  Search modes - Boolean/Phrase | Interface - EBSCOhost Research Databases  Search Screen - Advanced Search  Database - CINAHL Complete | Display |
| S25 | (MH "Home Health Aides") OR (MH "Community Medicine") | Expanders - Apply equivalent subjects  Search modes - Boolean/Phrase | Interface - EBSCOhost Research Databases  Search Screen - Advanced Search  Database - CINAHL Complete | Display |
| S26 | (MH "Physical Therapists") | Expanders - Apply equivalent subjects  Search modes - Boolean/Phrase | Interface - EBSCOhost Research Databases  Search Screen - Advanced Search  Database - CINAHL Complete | Display |
| S27 | (MH "Occupational Therapists") | Search modes - Boolean/Phrase | Interface - EBSCOhost Research Databases  Search Screen - Advanced Search  Database - CINAHL Complete | Display |
| S28 | TI community paramedic* OR AB community paramedic* | Search modes - Boolean/Phrase | Interface - EBSCOhost Research Databases  Search Screen - Advanced Search  Database - CINAHL Complete | Display |
| S29 | (MH "Telehealth") OR (MH "Telemedicine+") | Search modes - Boolean/Phrase | Interface - EBSCOhost Research Databases  Search Screen - Advanced Search  Database - CINAHL Complete | Display |
| S30 | TI ( telemedicine or telehealth or telecare ) OR AB ( telemedicine or telehealth or telecare ) | Search modes - Boolean/Phrase | Interface - EBSCOhost Research Databases  Search Screen - Advanced Search  Database - CINAHL Complete | Display |
| S31 | TI community OR AB ( community N2 (care or health*) ) OR TI ( community N2 (care or health*) ) | Search modes - Boolean/Phrase | Interface - EBSCOhost Research Databases  Search Screen - Advanced Search  Database - CINAHL Complete | Display |
| S32 | TI district N1 nurs* OR AB district N1 nurs* | Search modes - Boolean/Phrase | Interface - EBSCOhost Research Databases  Search Screen - Advanced Search  Database - CINAHL Complete | Display |
| S33 | S13 OR S14 OR S15 OR S16 OR S17 OR S18 OR S19 OR S20 OR S21 OR S22 OR S23 OR S24 OR S25 OR S26 OR S27 OR S28 OR S29 OR S30 OR S31 OR S32 | Search modes - Boolean/Phrase | Interface - EBSCOhost Research Databases  Search Screen - Advanced Search  Database - CINAHL Complete | Display |
| S34 | TI out of hours | Search modes - Boolean/Phrase | Interface - EBSCOhost Research Databases  Search Screen - Advanced Search  Database - CINAHL Complete | Display |
| S35 | (MH "Night Care") | Search modes - Boolean/Phrase | Interface - EBSCOhost Research Databases  Search Screen - Advanced Search  Database - CINAHL Complete | Display |
| S36 | TI ( ((outside or out or after or off) N2 (normal or working or office) N2 (time or hour*))) ) OR AB ( ((outside or out or after or off) N2 (normal or working or office) N2 (time or hour*))) ) | Search modes - Boolean/Phrase | Interface - EBSCOhost Research Databases  Search Screen - Advanced Search  Database - CINAHL Complete | Display |
| S37 | TI "after hour*" OR AB "after hour*" | Search modes - Boolean/Phrase | Interface - EBSCOhost Research Databases  Search Screen - Advanced Search  Database - CINAHL Complete | Display |
| S38 | TI out of hour* OR AB out of hour* | Search modes - Boolean/Phrase | Interface - EBSCOhost Research Databases  Search Screen - Advanced Search  Database - CINAHL Complete | Display |
| S39 | TI "off hour*" OR AB "off hour*" OR TI ( ((weekend* or evening* or holiday* or night*) N1 (hour* or care*)) ) OR AB ( ((weekend* or evening* or holiday* or night*) N1 (hour* or care*)) ) | Search modes - Boolean/Phrase | Interface - EBSCOhost Research Databases  Search Screen - Advanced Search  Database - CINAHL Complete | Display |
| S40 | TI ( ((24 hour* or 24H or around-the-clock or around the clock) N2 care*) ) OR AB ( ((24 hour* or 24H or around-the-clock or around the clock) N2 care*) ) | Search modes - Boolean/Phrase | Interface - EBSCOhost Research Databases  Search Screen - Advanced Search  Database - CINAHL Complete | Display |
| S41 | S34 OR S35 OR S36 OR S37 OR S38 OR S39 OR S40 | Expanders - Apply equivalent subjects  Search modes - Boolean/Phrase | Interface - EBSCOhost Research Databases  Search Screen - Advanced Search  Database - CINAHL Complete | Display |
| S42 | S12 or S33 OR S41 | Expanders - Apply equivalent subjects  Search modes - Boolean/Phrase | Interface - EBSCOhost Research Databases  Search Screen - Advanced Search  Database - CINAHL Complete | Display |
| S43 | S5 AND S42 | Expanders - Apply equivalent subjects  Search modes - Boolean/Phrase | Interface - EBSCOhost Research Databases  Search Screen - Advanced Search  Database - CINAHL Complete | Display |
| S44 | S12 AND S41 | Expanders - Apply equivalent subjects  Search modes - Boolean/Phrase | Interface - EBSCOhost Research Databases  Search Screen - Advanced Search  Database - CINAHL Complete | Display |
| S45 | S43 OR S44 | Expanders - Apply equivalent subjects  Search modes - Boolean/Phrase | Interface - EBSCOhost Research Databases  Search Screen - Advanced Search  Database - CINAHL Complete | Display |
| S46 | (TI (systematic* n3 review*)) or (AB (systematic* n3 review*)) or (TI (systematic* n3 bibliographic*)) or (AB (systematic* n3 bibliographic*)) or (TI (scoping n3 review)) or (AB (scoping n3 review)) or (TI (systematic* n3 literature)) or (AB (systematic* n3 literature)) or (TI (comprehensive* n3 literature)) or (AB (comprehensive* n3 literature)) or (TI (comprehensive* n3 bibliographic*)) or (AB (comprehensive* n3 bibliographic*)) or (TI (integrative n3 review)) or (AB (integrative n3 review)) or (JN “Cochrane Database of Systematic Reviews”) or (TI (information n2 synthesis)) or (TI (data n2 synthesis)) or (AB (information n2 synthesis)) or (AB (data n2 synthesis)) or (TI (data n2 extract*)) or (AB (data n2 extract*)) or (TI (medline or pubmed or psyclit or cinahl or (psycinfo not “psycinfo database”) or “web of science” or scopus or embase)) or (AB (medline or pubmed or psyclit or cinahl or (psycinfo not “psycinfo database”) or “web of science” or scopus or embase)) or (MH “Systematic Review”) or (MH “Meta Analysis”) or (TI (meta-analy* or metaanaly*)) or (AB (meta-analy* or metaanaly*)) | Search modes - Boolean/Phrase | Interface - EBSCOhost Research Databases  Search Screen - Advanced Search  Database - CINAHL Complete | Display |
| S47 | S45 AND S46 | Expanders - Apply equivalent subjects  Search modes - Boolean/Phrase | Interface - EBSCOhost Research Databases  Search Screen - Advanced Search  Database - CINAHL Complete | Display |

## Epistemonikos

(title:(palliat* OR hospice* OR "end of life" or “last year of life”) OR abstract:(palliat* OR hospice* OR "end of life" or “last year of life”)) AND (title:(community OR "primary care" OR "general practic*" OR "family practice" OR "district nurs*" OR home-based OR "at home" or “in the home” or “in-home” or “in home”OR "out of hours" OR OOH OR OOHs OR "after hours") OR abstract:(community OR "primary care" OR "general practic*" OR "family practice" OR "district nurs*" OR home-based OR "at home" OR "out of hours" OR OOH OR OOHs OR "after hours"))

# **Supplemental material 3: List of excluded studies**

| **Authors** | **Year** | **Title** | **Reason for exclusion** |
| --- | --- | --- | --- |
| Adams, M. | 2005 | Patient and carer satisfaction with palliative care services: a review of the literature | Wrong exposure |
| Afshar, K. and Geiger, K. and Muller-Mundt, G. and Bleidorn, J. and Schneider, N. | 2015 | [Generalist palliative care for non-cancer patients: A review article] | Wrong exposure |
| Agren Bolmsjo, I. | 2008 | End-of-life care for old people: a review of the literature | Wrong exposure |
| Almulla, H. & Hassouneh, D. | 2022 | Home-Based Palliative Care and Home Health Care in Saudi Arabia: An Integrative Literature Review | Wrong exposure |
| Alshakhs, S., Park, T., McDarby, M., Reid, M. C., Czaja, S., Adelman, R., Sweet, E., Jedlicka, C. M., Delgado, D. & Phongtankuel, V. | 2023 | Interventions for Family Caregivers of Patients Receiving Palliative/Hospice Care at Home: A Scoping Review | Wrong exposure |
| Amin A, Besdine RK, Blazer DG, Cohen H, Fulmer T, Ganz PA, Grunwald M, Hall WJ, Katz PR, Kitzman DW | 2007 | Assessing care of vulnerable elders-3 quality indicators | Wrong study type |
| Aoun, S. M. and Breen, L. J. and Howting, D. | 2014 | The support needs of terminally ill people living alone at home: a narrative review | Wrong exposure |
| Arias Rojas, M. and García-Vivar, C. | 2015 | The transition of palliative care from the hospital to the home: a narrative review of experiences of patients and family caretakers | Wrong exposure |
| Bachnick, S. | 2014 | Palliative care in family life: a systematic literature review about unmet needs | Wrong language |
| Bakitas, M. A. and Elk, R. and Astin, M. and Ceronsky, L. and Clifford, K. N. and Dionne-Odom, J. N. and Emanuel, L. L. and Fink, R. M. and Kvale, E. and Levkoff, S. and Ritchie, C. and Smith, T. | 2015 | Systematic Review of Palliative Care in the Rural Setting | Wrong exposure |
| Baumbach, A., Hughes, M. C., Liu, Y. | 2024 | Challenges and Coping Strategies in Transitioning From Caregiving to Widowhood: A Systematic Review | Wrong population |
| Bayly J, Bone AE, Ellis-Smith C, Yaqub S, Yi D, Nkhoma KB, Cook A, Combes S, Bajwah S, Harding R, Nicholson C. | 2021 | Common elements of service delivery models that optimise quality of life and health service use among older people with advanced progressive conditions: a tertiary systematic review | Wrong exposure |
| Beasley, A. and Bakitas, M. A. and Edwards, R. and Kavalieratos, D. | 2019 | Models of non-hospice palliative care: a review | Wrong exposure |
| Beattie, J. M. and Johnson, M. J. | 2012 | Subcutaneous furosemide in advanced heart failure: has clinical practice run ahead of the evidence base? | Wrong study type |
| Becqué, Y. N., van der Wel, M., Aktan-Arslan, M., Geert van Driel, A., Rietjens, J. A. C., van der Heide, A. & Witkamp, E. | 2023 | Supportive interventions for family caregivers of patients with advanced cancer: A systematic review | Wrong exposure |
| Bee, P. E. and Barnes, P. and Luker, K. A. | 2009 | A systematic review of informal caregivers' needs in providing home-based end-of-life care to people with cancer | Wrong exposure |
| Benitez, M. A. and Castaneda, P. and Gimeno, V. and Gomez, M. and Duque, A. and Pascual, L. and Perez, N. and Sanchez, M. and Torrubia, P. and semFYC, Secpal | 2001 | [Consensus document of the Spanish Society of Palliative Care (SECPAL) and the Spanish Society of Family and Community Medicine (semFYC). Domiciliary care for the patient with cancer in terminal phase] | Wrong study type |
| Bergen A. | 1991 | Nurses caring for the terminally ill in the community: a review of the literature | Wrong population |
| Bergin, S. and Mockford, C. | 2016 | Recommendations to support informal carers of people living with motor neurone disease | Wrong exposure |
| Boland, Laura, Perez, Maria Margarita Becerra, Menear, Matthew, Garvelink, Mirjam M., McIsaac, Daniel I., Guérard, Geneviève Painchaud, Emond, Julie, Brière, Nathalie, Stacey, Dawn | 2017 | Impact of home care versus alternative locations of care on elder health outcomes: an overview of systematic reviews | Wrong population |
| Bolt, S. R. and van der Steen, J. T. and Schols, Jmga and Zwakhalen, S. M. G. and Pieters, S. and Meijers, J. M. M. | 2019 | Nursing staff needs in providing palliative care for people with dementia at home or in long-term care facilities: A scoping review | Wrong exposure |
| Brazil, K. and Howell, D. and Marshall, D. and Critchley, P. and Van den Elzen, P. and Thomson, C. | 2007 | Building primary care capacity in palliative care: proceedings of an interprofessional workshop | Wrong study type |
| Burns, J. and Polus, S. and Brereton, L. and Chilcott, J. and Ward, S. E. and Pfadenhauer, L. M. and Rehfuess, E. A. | 2018 | Looking beyond the forest: Using harvest plots, gap analysis, and expert consultations to assess effectiveness, engage stakeholders, and inform policy | Wrong study type |
| Cameron, D. and Johnston, B. | 2015 | Development of a questionnaire to measure the key attributes of the community palliative care specialist nurse role | Wrong exposure |
| Candy, B. and France, R. and Low, J. and Sampson, L. | 2015 | Does involving volunteers in the provision of palliative care make a difference to patient and family wellbeing? A systematic review of quantitative and qualitative evidence | Wrong exposure |
| Carcavilla-Gonzalez, N., Escalada San Adrian, G., Minobes-Molina, E., Pamies-Tejedor, S., Roncal-Belzunce, V., Atares-Rodriguez, L., Garcia-Navarro, J. A. | 2024 | A Paradigm Shift on Deinstitutionalization and Dementia Care: A Narrative Review | No full text available |
| Carey, M. L. and Zucca, A. C. and Freund, M. A. and Bryant, J. and Herrmann, A. and Roberts, B. J. | 2019 | Systematic review of barriers and enablers to the delivery of palliative care by primary care practitioners | Wrong exposure |
| Chambers, J. | 2008 | In-home palliative care increased patient satisfaction and reduced use and costs of medical services | Wrong study type |
| Chan, R. J. and Webster, J. and Bowers, A. | 2016 | End-of-life care pathways for improving outcomes in caring for the dying | Wrong exposure |
| Chen, L., Sleeman, K. E., Bradshaw, A., Sakharang, W., Mo, Y., Ellis-Smith, C. | 2024 | The Use of Person-Centered Outcome Measures to Support Integrated Palliative Care for Older People: A Systematic Review | Wrong exposure |
| Chi, N. C. and Demiris, G. | 2015 | A systematic review of telehealth tools and interventions to support family caregivers | Wrong population |
| Chong, P. H. and Yeo, Z. Z. | 2021 | Parenteral Lidocaine for Complex Cancer Pain in the Home or Inpatient Hospice Setting: A Review and Synthesis of the Evidence | Wrong study type |
| Christodoulou, L., Parpottas, P., Petkari, E. | 2024 | Psychological interventions to enhance positive outcomes in adult cancer caregivers: A systematic review | Wrong exposure |
| Chung, A. and Collier, A. and Gott, M. | 2019 | Community-led and/or focused initiatives to support family carers within a palliative care context: An integrative review | Wrong exposure |
| Clyne, B. and O'Neill, S. M. and Nuzum, D. and O'Neill, M. and Larkin, J. and Ryan, M. and Smith, S. M. | 2019 | Patients' spirituality perspectives at the end of life: a qualitative evidence synthesis | Wrong exposure |
| Coelho, A. and Parola, V. and Cardoso, D. and Bravo, M. E. and Apostolo, J. | 2017 | Use of non-pharmacological interventions for comforting patients in palliative care: a scoping review | Wrong exposure |
| Coelho, A. and Parola, V. and Cardoso, D. and Escobar, M. and Apostolo, J. | 2016 | The use of non-pharmacological interventions for the comfort of patients in palliative care: A scoping review protocol | Wrong study type |
| Collins, Anna and Brown, Julia E. H. and Mills, Jason and Philip, Jennifer | 2021 | The impact of public health palliative care interventions on health system outcomes: A systematic review | Wrong exposure |
| Cotton, A. H. and Sayers, J. M. | 2016 | Older persons' perceptions and experiences of community palliative care: a systematic review of qualitative evidence protocol | Wrong exposure |
| Cowley, A. and Bath-Hextall, F. and Cooper, J. | 2017 | Interventions for healthcare professionals, organizations and patients to enhance quality of life for people diagnosed with palliative esophagogastric cancer: a systematic review | Wrong exposure |
| Cowley, A. and Evans, C. and Bath-Hextall, F. and Cooper, J. | 2016 | Patient, nursing and medical staff experiences and perceptions of the care of people with palliative esophagogastric cancer: a systematic review of the qualitative evidence | Wrong exposure |
| Crimmins, R. M. and Elliott, L. and Absher, D. T. | 2021 | Palliative Care in a Death-Denying Culture: Exploring Barriers to Timely Palliative Efforts for Heart Failure Patients in the Primary Care Setting | Wrong exposure |
| Cruz, S., Fernandes, C. & Magalhaes, B. | 2023 | A scoping review of mobile apps for use with palliative patients in the context of home care | Wrong exposure |
| Das, D. and Ali, M. and Hussain, I. A. and Ingram, J. T. N. and Johnstone, R. S. and Lopes, J. J. and Wadee, T. and Chakraborty, N. | 2021 | What do we know about patients' perspectives and expectations relating to palliative and end-of-life care in advanced liver disease? A systematic review of qualitative literature using ENTREQ guidelines | Wrong exposure |
| Davis, E. M. and Jamison, P. and Brumley, R. and Enguidanos, S. | 2006 | Barriers and facilitators to replicating an evidence-based palliative care model | Wrong study type |
| Despotova-Toleva L., and Toleva-Nowak N. | 2021 | Narrative review of home care for palliative patients in general practice | Wrong exposure |
| Devlin, M. and McIlfatrick, S. | 2009 | The role of the home-care worker in palliative and end-of-life care in the community setting: a literature review | Wrong study type |
| Dias Franҫa, Bruna and Silva, Kênia Lara and de Araújo Ferreira, Jocelly and Silva, Alexandre Ernesto and das Chagas Cunha Gonҫalves Neta, Francisca | 2019 | Perception of patients about palliative care | Wrong exposure |
| DiMartino, L. D. and Weiner, B. J. and Mayer, D. K. and Jackson, G. L. and Biddle, A. K. | 2014 | Do palliative care interventions reduce emergency department visits among patients with cancer at the end of life? A systematic review | Wrong exposure |
| Disler, R. and Jones, A. | 2010 | District nurse role in end-stage COPD: a review | Wrong exposure |
| Dolan, H. and Eggett, C. and Holliday, L. and Delves, S. and Parkes, D. and Sutherland, K. | 2021 | Virtual care in end of life and palliative care: A rapid evidence check | Wrong population |
| Ebneter, A. S. and Sauter, T. C. and Christen, A. and Eychmueller, S. | 2022 | Feasibility, acceptability and needs in telemedicine for palliative care | Wrong exposure |
| Edwards, S. T. and Peterson, K. and Chan, B. and Anderson, J. and Helfand, M. | 2017 | Effectiveness of Intensive Primary Care Interventions: A Systematic Review | Wrong exposure |
| ElMokhallalati, Y. and Bradley, S. H. and Chapman, E. and Ziegler, L. and Murtagh, F. E. and Johnson, M. J. and Bennett, M. I. | 2020 | Identification of patients with potential palliative care needs: A systematic review of screening tools in primary care | Wrong exposure |
| Eriki, P. | 2020 | Home-based care for patients with COVID-19 | Wrong exposure |
| Evans CJ, Ison L, Ellis‐smith CL, Nicholson C, Costa A, Oluyase AO, Namisango E, Bone AE, Brighton LJ, Yi D, Combes S. | 2019 | Service Delivery Models to Maximize Quality of Life for Older People at the End of Life: A Rapid Review | Wrong exposure |
| Evans, Catherine J., Ison, Lucy, Ellis-Smith, Clare, Nicholson, Caroline, Costa, Alessia, Oluyase, Adejoke O., Namisango, Eve, Bone, Anna E., Brighton, Lisa Jane, Yi, Deokhee, Combes, Sarah, Bajwah, Sabrina, Gao, Wei, Harding, Richard, Ong, Paul, Higginson, Irene J., Maddocks, Matthew | 2019 | Service Delivery Models to Maximize Quality of Life for Older People at the End of Life: A Rapid Review | Wrong exposure |
| Fang, Shuyan, Zhi, Shengze, Song, Dongpo, Sun, Juanjuan, Gao, Shizheng, Wang, Yonghong, Sun, Jiao, Dong, Wanhui | 2024 | Dignity-preserving care of people with dementia in different nursing environments: a qualitative systematic review | No full text available |
| Fee, A. and Muldrew, D. and Slater, P. and Payne, S. and McIlfatrick, S. and McConnell, T. and Finlay, D. A. and Hasson, F. | 2020 | The roles, responsibilities and practices of healthcare assistants in out-of-hours community palliative care: A systematic scoping review | Wrong exposure |
| Foster, H. and Moffat, K. R. and Burns, N. and Gannon, M. and Macdonald, S. and O'Donnell, C. A. | 2020 | What do we know about demand, use and outcomes in primary care out-of-hours services? A systematic scoping review of international literature | Wrong population |
| Francis, S. A., Yardley, S., Franklin, B., Ogden, M., Kajamaa, A., Mattick, K. | 2023 | A model of intended and ideal prescribing and medication use in symptom control in palliative care: an international scoping review | No full text available |
| Fuhrman, M. P. and Galvin, T. A. and Ireton-Jones, C. S. and Thorpe, J. and American Dietetic, Association | 2009 | Practice paper of the American Dietetic Association: Home care--opportunities for food and nutrition professionals | Wrong study type |
| Gadoud, A. C. and Johnson, M. J. | 2014 | Response: what tools are available to identify patients with palliative care needs in primary care: a systematic literature review and survey of European practice? | Wrong study type |
| Gans, D. and Ganz, D. A. and Senelick, W. and McCreath, H. E. and Jew, J. and Osterweil, D. and Batra, R. A. and Tan, Z. and Jennings, L. A. and Reuben, D. B. | 2016 | A Strategy for Identifying and Disseminating Best Practice Innovations in the Care of Patients with Multiple Chronic Conditions or End-of-Life Care Needs | Wrong study type |
| Gardiner, C. and Ingleton, C. and Ryan, T. and Ward, S. and Gott, M. | 2017 | What cost components are relevant for economic evaluations of palliative care, and what approaches are used to measure these costs? A systematic review | Wrong exposure |
| Gill, A. and Laporte, A. and Coyte, P. C. | 2013 | Predictors of home death in palliative care patients: a critical literature review | Wrong exposure |
| Golinelli, Davide, Boetto, Erik, Carullo, Gherardo, Nuzzolese, Andrea Giovanni, Landini, Maria Paola, Fantini, Maria Pia | 2020 | Adoption of Digital Technologies in Health Care During the COVID-19 Pandemic: Systematic Review of Early Scientific Literature | Wrong exposure |
| Goodman, C. and Evans, C. and Wilcock, J. and Froggatt, K. and Drennan, V. and Sampson, E. and Blanchard, M. and Bissett, M. and Iliffe, S. | 2010 | End of life care for community dwelling older people with dementia: an integrated review | Wrong exposure |
| Goodwin, N., Lewis, S., Curryer, C., Triandafilidis, Z., Jeong, S., Carr S. & Davis, D. | 2022 | Models of End of Life Care to Support People with Dementia: Results of an Evidence Review | Wrong study type |
| Goossensen, Anne and Somsen, Jos and Scott, Ros and Pelttari, Leena | 2016 | Defining volunteering in hospice and palliative care in Europe: an EAPC White Paper | Wrong exposure |
| Gorst, Sarah L., Armitage, Christopher J., Brownsell, Simon, Hawley, Mark S. | 2014 | Home telehealth uptake and continued use among heart failure and chronic obstructive pulmonary disease patients: a systematic review | Wrong population |
| Gruenewald, D. A., Higginson, Irene J., Vivat, Bella, Edmonds, Polly, Burman, Rachel | 2004 | Quality of life measures for the palliative care of people severely affected by multiple sclerosis: a systematic review | Wrong exposure |
| Guilbeau, C. | 2018 | End-of-life care in the Western world: where are we now and how did we get here? | Wrong exposure |
| Harding, R. and Higginson, I. J. | 2003 | What is the best way to help caregivers in cancer and palliative care? A systematic literature review of interventions and their effectiveness | Wrong population |
| Harding, R. and Karus, D. and Easterbrook, P. and Raveis, V. H. and Higginson, I. J. and Marconi, K. | 2005 | Does palliative care improve outcomes for patients with HIV/AIDS? A systematic review of the evidence | Wrong exposure |
| Haroen, H., Harun, H., Sari, C. W. M., Witdiawati, W. | 2023 | Uncovering Methods and Outcomes of Palliative Care for Geriatric Patients: A Scoping Review | Wrong exposure |
| Harris, Iain, Murray, Scott A. | 2013 | Can palliative care reduce futile treatment? A systematic review | Wrong exposure |
| Hashem, F. and Brigden, C. and Wilson, P. and Butler, C. | 2020 | Understanding what works, why and in what circumstances in hospice at home services for end-of-life care: Applying a realist logic of analysis to a systematically searched literature review | Wrong exposure |
| Hasson, F. and Betts, M. and Shannon, C. and Fee, A. | 2022 | Roles and responsibilities of the community palliative care key worker: a scoping review | Wrong population |
| Hatzikiriakidis, K., Darshini, A., Skouteris, H., Patitsas, H., Smith, K., Dhulia, A. & Poon, P. | 2023 | A rapid umbrella review of the literature surrounding the provision of patient-centred end-of-life care | Wrong exposure |
| Hearn, J. and Higginson, I. J. | 1999 | Development and validation of a core outcome measure for palliative care: the palliative care outcome scale. Palliative Care Core Audit Project Advisory Group | Wrong study type |
| Herber, O. R. and Johnston, B. M. | 2013 | The role of healthcare support workers in providing palliative and end-of-life care in the community: a systematic literature review | Wrong population |
| Higginson, I. J. and Evans, C. J. | 2010 | What is the evidence that palliative care teams improve outcomes for cancer patients and their families? | Wrong exposure |
| Higginson, I. J. and Finlay, I. G. and Goodwin, D. M. and Hood, K. and Edwards, A. G. and Cook, A. and Douglas, H. R. and Normand, C. E. | 2003 | Is there evidence that palliative care teams alter end-of-life experiences of patients and their caregivers? | Wrong exposure |
| Hill, A. | 2001 | Community palliative care: the evolving role of Macmillan nurses | Wrong study type |
| Hirdes, J. P. and Freeman, S. and Smith, T. F. and Stolee, P. | 2012 | Predictors of caregiver distress among palliative home care clients in Ontario: evidence based on the interRAI Palliative Care | Wrong study type |
| Ho, Ge Kai and Chye, Richard and Jang, Danny and Sutton, Patricia and Sullivan, Sandra and Seah, Davinia | 2020 | Factors Associated with Re-Enrollment of Patients from a Specialist Community Palliative Care Service | Wrong study type |
| Hojjat-Assari, S. and Rassouli, M. and Madani, M. and Heydari, H. | 2021 | Developing an integrated model of community-based palliative care into the primary health care (PHC) for terminally ill cancer patients in Iran | Wrong study type |
| Hospice Association of South, Africa | 2001 | Palliative care | Wrong study type |
| Hudson, P. and Collins, A. and Bostanci, A. and Willenberg, L. and Stepanov, N. and Philip, J. | 2016 | Toward a systematic approach to assessment and care planning in palliative care: A practical review of clinical tools | Wrong exposure |
| Huggins, M., Pesut, B. & Puurveen, G. | 2023 | Interventions for Caregivers of Older Adults with Dementia Living in the Community: A Rapid Review of Reviews | Wrong population |
| Hussainy, S. Y. and Box, M. and Scholes, S. | 2011 | Piloting the role of a pharmacist in a community palliative care multidisciplinary team: An Australian experience | Wrong study type |
| Hyunjin, Noh and Paradiso de Sayu, Rebecca and Granda Anderson, Kimberly and Ford, Cassandra D. | 2016 | Community-Based Participatory Research on Issues Around Palliative and End-of-Life Care | Wrong exposure |
| Jang, J. and Lazenby, M. | 2013 | Current state of palliative and end-of-life care in home versus inpatient facilities and urban versus rural settings in Africa | Wrong exposure |
| Jie li, wuga jishi |  | Home-based palliative care in heart failure&#xff1a; a meta-analysis of RCT | No full text available |
| Johnston, B. M. and McCauley, R. and McQuillan, R. and Rabbitte, M. and Honohan, C. and Mockler, D. and Thomas, S. and May, P. | 2020 | Effectiveness and cost-effectiveness of out-of-hours palliative care: a systematic review | Wrong exposure |
| Johnston, Bridget M. and McCauley, Rachel and Rabbitte, Mary and McQuillan, Regina and Honohan, Caitriona and Mockler, David and Thomas, Steve and May, Peter | 2021 | Evidence on the effectiveness and cost-effectiveness of out-of-hours palliative care | Wrong study type |
| Jordan, R. I. and Allsop, M. J. and ElMokhallalati, Y. and Jackson, C. E. and Edwards, H. L. and Chapman, E. J. and Deliens, L. and Bennett, M. I. | 2020 | Duration of palliative care before death in international routine practice: a systematic review and meta-analysis | Wrong exposure |
| Katz, M. | 2020 | Telehealth increases access to palliative care for people with Parkinson's disease and related disorders | Wrong exposure |
| Kearns, T. and Cornally, N. and Molloy, W. | 2017 | Patient reported outcome measures of quality of end-of-life care: A systematic review | Wrong exposure |
| Keeley, D. | 1999 | Rigorous assessment of palliative care revisited. Wisdom and compassion are needed when evidence is lacking | Wrong study type |
| Keenan, AnnMarie and Keithley, Joyce K. | 2015 | Integrative Review: Effects of Music on Cancer Pain in Adults | Wrong exposure |
| Kelley, L. T. and Coderre-Ball, A. M. and Dalgarno, N. and McKeown, S. and Egan, R. | 2020 | Continuing Professional Development for Primary Care Providers in Palliative and End-of-Life Care: A Systematic Review | Wrong exposure |
| Kerr, H. and Price, J. and Nicholl, H. and O'Halloran, P. | 2017 | Transition from children's to adult services for young adults with life-limiting conditions: A realist review of the literature | Wrong exposure |
| Killackey, T. and Lovrics, E. and Saunders, S. and Isenberg, S. R. | 2020 | Palliative care transitions from acute care to community-based care: A qualitative systematic review of the experiences and perspectives of health care providers | Wrong exposure |
| Kim, S. L. and Tarn, D. M. | 2016 | Effect of Primary Care Involvement on End-of-Life Care Outcomes: A Systematic Review | Wrong exposure |
| Kirby, Sue and Barlow, Veronica and Saurman, Emily and Lyle, David and Passey, Megan and Currow, David | 2016 | Are rural and remote patients, families and caregivers needs in life-limiting illness different from those of urban dwellers? A narrative synthesis of the evidence | Wrong exposure |
| Klein, S. and Hostetter, M. and McCarthy, D. | 2017 | An Overview of Home-Based Primary Care: Learning from the Field | Wrong study type |
| Kreyer, C. and Pleschberger, S. | 2014 | [Struggling for normal in an instable situation - informal caregivers self-management in palliative home care. A meta-synthesis] | Wrong population |
| Kristanti, M. S., Agastiya, I. M. C. & Kurianto, E. | 2023 | The Implementation of Palliative Home Care in Southeast Asian Countries: An Integrative Review | Wrong exposure |
| Latif, Aulia Insani and Irwan, Andi Masyitha | 2019 | Models and benefits of palliative care for the quality of life of people with HIV: a systematic review | No full text available |
| Latorraca, C. O. and Martimbianco, A. L. C. and Pachito, D. V. and Torloni, M. R. and Pacheco, R. L. and Pereira, J. G. and Riera, R. | 2019 | Palliative care interventions for people with multiple sclerosis | Wrong exposure |
| Leclerc-Loiselle, J., Gendron, S., Daneault, S. | 2024 | Nursing activities for health promotion in palliative home care: an integrative review | Wrong population |
| Lewis, S., Triandafilidis, Z., Curryer, C., Jeong, S. Y., Goodwin, N., Carr, S. & Daneill, D. | 2023 | Models of care for people with dementia approaching end of life: A rapid review | Wrong exposure |
| Librada-Flores, S. and Nabal-Vicuna, M. and Forero-Vega, D. and Munoz-Mayorga, I. and Guerra-Martin, M. D. | 2020 | Implementation Models of Compassionate Communities and Compassionate Cities at the End of Life: A Systematic Review | Wrong exposure |
| Liu, H., Lou, V. W. Q., Xu, S. | 2024 | Randomized controlled trials on promoting self-care behaviors among informal caregivers of older patients: a systematic review and meta-analysis | Wrong population |
| Lo, B. and Quill, T. and Tulsky, J. | 1999 | Discussing palliative care with patients. ACP-ASIM End-of-Life Care Consensus Panel. American College of Physicians-American Society of Internal Medicine | Wrong study type |
| Lowe, S. S. and Watanabe, S. M. and Baracos, V. E. and Courneya, K. S. | 2013 | Home-based functional walking program for advanced cancer patients receiving palliative care: a case series | Wrong study type |
| Lundereng, E. D. and Nes, A. A. G. and Holmen, H. and Winger, A. and Thygesen, H. and Joranson, N. and Borge, C. R. and Chen, W. and Dajani, O. and Mariussen, K. L. and Steindal, S. A. | 2021 | Health Care Professionals' Experiences and Perspectives on Using Telehealth for Home-Based Palliative Care: Protocol for a Scoping Review | Wrong study type |
| Mackinnon, C. J. | 2009 | Applying feminist, multicultural, and social justice theory to diverse women who function as caregivers in end-of-life and palliative home care | Wrong population |
| MacRae, M. C. and Fazal, O. and O'Donovan, J. | 2020 | Community health workers in palliative care provision in low-income and middle-income countries: a systematic scoping review of the literature | Wrong population |
| Mandel, M. and Savoy, E. | 2011 | Research on supporting patients and family caregivers with palliative and end-of-life care | Wrong study type |
| Maradana, S. and Kate, Y. and Pandey, D. and Syed, M. P. and Velamala, P. R. and Patil, S. and Siddiqui, A. D. | 2020 | Barriers to goals of care discussion and early palliative care referral in patients with advanced cancer: A community oncology clinic survey | Wrong study type |
| Margaret Dunham, Karen Campbell Maria King |  | A systematic review to identify the use of telehealth initiatives to support remote communication and facilitate home-based specialist palliative care and end of life (hospice) services for adults | No full text available |
| Markgren, R. and Brannstrom, M. and Lundgren, C. and Boman, K. | 2019 | Impacts of person-centred integrated chronic heart failure and palliative home care on pharmacological heart failure treatment: a substudy of a randomised trial | Wrong study type |
| Marsella, A. | 2009 | Exploring the literature surrounding the transition into palliative care: a scoping review | Wrong exposure |
| Marsh, Pauline and Spinaze, Anna | 2016 | Community gardens as sites of solace and end-of-life support: a literature review | Wrong exposure |
| Martin, J. L. and Saredakis, D. and Hutchinson, A. D. and Crawford, G. B. and Loetscher, T. | 2022 | Virtual Reality in Palliative Care: A Systematic Review | Wrong exposure |
| Martinez-Gonzalez, N. A. and Coenen, S. and Plate, A. and Colliers, A. and Rosemann, T. and Senn, O. and Neuner-Jehle, S. | 2017 | The impact of interventions to improve the quality of prescribing and use of antibiotics in primary care patients with respiratory tract infections: a systematic review protocol | Wrong study type |
| Mathew, C. and Hsu, A. T. and Prentice, M. and Lawlor, P. and Kyeremanteng, K. and Tanuseputro, P. and Welch, V. | 2020 | Economic evaluations of palliative care models: A systematic review | Wrong exposure |
| Matis, J. and Svetlak, M. and Slezackova, A. and Svoboda, M. and Sumec, R. | 2020 | Mindfulness-Based Programs for Patients With Cancer via eHealth and Mobile Health: Systematic Review and Synthesis of Quantitative Research | Wrong exposure |
| McDarby, Meghan and Llaneza, Danielle and George, Login and Kozlov, Elissa | 2021 | Mobile Applications for Advance Care Planning: A Comprehensive Review of Features, Quality, Content, and Readability | Wrong exposure |
| McPherson, C. J. | 2021 | Qualitative synthesis of research on healthcare providers' perceptions of the transition from in-patient to community palliative care | Wrong exposure |
| McPherson, C. J. and Etele, J. and Ta, V. C. and Raghubir, A. | 2019 | Unregulated care providers' engagement in palliative care to older clients and their families in the home setting: a mixed methods study | Wrong study type |
| Mercadante, S. and Porzio, G. and Valle, A. and Fusco, F. and Aielli, F. and Costanzo, V. and Home Care Italy, Group | 2011 | Palliative sedation in patients with advanced cancer followed at home: a systematic review | Wrong exposure |
| Miller, E. M. and Porter, J. E. | 2021 | Understanding the Needs of Australian Carers of Adults Receiving Palliative Care in the Home: A Systematic Review of the Literature | Wrong population |
| Mills, K. and Payne, A. | 2015 | Enabling occupation at the end of life: A literature review | Wrong exposure |
| Mitchell, Geoffrey, Burridge, Letitia, Zhang, Jianzhen, Donald, Maria, Scott, Ian A., Dart, J. M., Jackson, Claire | 2015 | Systematic review of integrated models of health care delivered at the primary-secondary interface: how effective is it and what determines effectiveness? | Wrong population |
| Mitchell, S. and Maynard, V. and Lyons, V. and Jones, N. and Gardiner, C. | 2020 | The role and response of primary healthcare services in the delivery of palliative care in epidemics and pandemics: A rapid review to inform practice and service delivery during the COVID-19 pandemic | Wrong exposure |
| Mo, L. and Hui, D. | 2021 | Reply to comment on: Referral criteria to specialist palliative care for patients with dementia-A systematic review | Wrong study type |
| Mojtahedi, Z. and Shen, J. J. | 2022 | Home Palliative Care during the COVID-19 Pandemic: A Scoping Review | Wrong study type |
| Morris, S. M. and King, C. and Turner, M. and Payne, S. | 2015 | Family carers providing support to a person dying in the home setting: A narrative literature review | Wrong exposure |
| Moynihan, R. | 2012 | Reasons to be hopeful: streams of renewal in healthcare | Wrong study type |
| Murray, M. A. and Fiset, V. and Young, S. and Kryworuchko, J. | 2009 | Where the dying live: A systematic review of determinants of place of end-of-life cancer care | Wrong exposure |
| Murray, S. A. and Firth, A. and Schneider, N. and Van den Eynden, B. and Gomez-Batiste, X. and Brogaard, T. and Villanueva, T. and Abela, J. and Eychmuller, S. and Mitchell, G. and Downing, J. and Sallnow, L. and van Rijswijk, E. and Barnard, A. and Lynch, M. and Fogen, F. and Moine, S. | 2015 | Promoting palliative care in the community: production of the primary palliative care toolkit by the European Association of Palliative Care Taskforce in primary palliative care | Wrong exposure |
| Murray, S. A. and Mitchell, G. K. and Burge, F. and Barnard, A. and Nowels, D. and Charlton, R. | 2006 | It's time to develop primary care services for the dying | Wrong study type |
| Nabukalu, D., Gordon, L. G., Lowe, J., Merollini, K. M. D. | 2024 | Healthcare costs of cancer among children, adolescents, and young adults: A scoping review | Wrong population |
| Nadin, Shevaun and Crow, Maxine and Prince, Holly and Kelley, Mary Lou | 2018 | Wiisokotaatiwin: development and evaluation of a community-based palliative care program in Naotkamegwanning First Nation | Wrong exposure |
| Namasivayam, P. and Bui, D. T. and Low, C. and Barnett, T. and Bridgman, H. and Marsh, P. and Lee, S. | 2022 | Use of telehealth in the provision of afterhours palliative care services in rural and remote Australia: A scoping review protocol | Wrong study type |
| Naoum P., Pavi E., and Athanasakis K. | 2021 | Economic Evaluation of Digital Health Interventions in Palliative Care: A Systematic Review of the Literature | Wrong population |
| Ngwenya, N. and Kenten, C. and Jones, L. and Gibson, F. and Pearce, S. and Flatley, M. and Hough, R. and Stirling, L. C. and Taylor, R. M. and Wong, G. and Whelan, J. | 2017 | Experiences and Preferences for End-of-Life Care for Young Adults with Cancer and Their Informal Carers: A Narrative Synthesis | Wrong exposure |
| Nordennen, Ronald and Lavrijsen, Jan and Vissers, Kris and Koopmans, Raymond | 2014 | Decision Making About Change of Medication for Comorbid Disease at the End of Life: An Integrative Review | Wrong exposure |
| Ogi, M. and Campling, N. and Birtwistle, J. and Richardson, A. and Bennett, M. I. and Santer, M. and Latter, S. | 2021 | Community access to palliative care medicines-patient and professional experience: systematic review and narrative synthesis | Wrong exposure |
| O'Hanlon, C. E. and Walling, A. M. and Okeke, E. and Stevenson, S. and Wenger, N. S. | 2018 | A Framework to Guide Economic Analysis of Advance Care Planning | Wrong exposure |
| Oishi, A. and Murtagh, F. E. | 2014 | The challenges of uncertainty and interprofessional collaboration in palliative care for non-cancer patients in the community: a systematic review of views from patients, carers and health-care professionals | Wrong exposure |
| Olano â€ Lizarraga, Maddi and Oroviogoicoechea, Cristina and Errasti â€ Ibarrondo, BegoÃ±a and SaracÃ­bar â€ Razquin, Maribel | 2016 | The personal experience of living with chronic heart failure: a qualitative meta-synthesis of the literature | Wrong exposure |
| Oldham, L. and Kristjanson, L. J. | 2004 | Pain management education for family carers of people living with advanced cancer in the community | Wrong study type |
| Oliveira, Stefanie Griebeler and Quintana, Alberto Manuel and Budo, Maria de Lourdes Denardin and Ludtke, Manoela Fonseca and Cassel, Paula Argemi and Wottrich, Shana Hastenpflug | 2011 | Family caregivers and end-of-life: tendencies of the scientific production in the health field | Wrong population |
| Park, E. J. and Jo, M. and Park, M. and Kang, S. J. | 2021 | Advance care planning for older adults in community-based settings: An umbrella review | Wrong exposure |
| Parola, V. and Coelho, A. and Neves, H. and Teixeira, J. and Lima, A. and Ferreira, J. and Cruz, A. | 2021 | Palliative rehabilitation interventions in palliative care: a scoping review. [Italian] | Wrong language |
| Pastor, D. K. and Moore, G. | 2013 | Uncertainties of the heart: palliative care and adult heart failure | Wrong exposure |
| Patton L., Avsar P., Nugent P., O'Connor T., Patton D., Moore Z. | 2021 | What is the impact of specialist palliative care outpatient consultations on pain in adult patients with cancer? A systematic review | Wrong exposure |
| Payne, S. and Chapman, A. and Holloway, M. and Seymour, J. E. and Chau, R. and Payne, Sheila and Chapman, Alice and Holloway, Margaret and Seymour, Jane E. and Chau, Ruby | 2005 | Chinese community views: Promoting cultural competence in palliative care | Wrong exposure |
| Pesut, B. and Hooper, B. and Sawatzky, R. and Robinson, C. A. and Bottorff, J. L. and Dalhuisen, M. | 2013 | Program assessment framework for a rural palliative supportive service | Wrong study type |
| Petrova, M. and Wong, G. and Kuhn, I. and Wellwood, I. and Barclay, S. | 2021 | Timely community palliative and end-of-life care: a realist synthesis | Wrong study type |
| Pham, B. and Krahn, M. | 2014 | End-of-Life Care Interventions: An Economic Analysis | Wrong exposure |
| Pollock, K. and Wilson, E. | 2015 | Care and communication between health professionals and patients affected by severe or chronic illness in community care settings: a qualitative study of care at the end of life (Structured abstract) | Wrong study type |
| Poornima, Sunder and Shoba, Nair and Rajashree, K. Chittazhathu and Athul Joseph, Manuel and Biju, Raghavan and Sunil, M. M. Kumar and Vineetha, Rijju and Geetha, Vijay and Seema, Rao and Anupama, V. Prabhu and Mhoira, Leng and Rajagopal, M. R. and Odette, Spruijt and Uma, Parameswaran and Sunitha, Daniel and Chitra, Venkateswaran | 2020 | Responding to Palliative Care Training Needs in the Coronavirus Disease 2019 Era: The Context and Process of Developing and Disseminating Training Resources and Guidance for Low- and Middle-Income Countries from Kerala, South India | Wrong study type |
| Poudel, A. and Yates, P. and Rowett, D. and Nissen, L. M. | 2017 | Use of Preventive Medication in Patients With Limited Life Expectancy: A Systematic Review | Wrong exposure |
| Putranto, D. and Rochmawati, E. | 2020 | Mobile applications for managing symptoms of patients with cancer at home: A scoping review | Wrong exposure |
| Qi He Mabel, L. and Drury, V. B. and Hong, P. W. | 2010 | The experience and expectations of terminally ill patients receiving music therapy in the palliative setting: a systematic review | Wrong exposure |
| Ramasamy Venkatasalu, M. and Sirala Jagadeesh, N. and Elavally, S. and Pappas, Y. and Mhlanga, F. and Pallipalayam Varatharajan, R. | 2018 | Public, patient and carers' views on palliative and end-of-life care in India | Wrong exposure |
| Raunkiaer, M. and Timm, H. | 2013 | Interventions concerning competence building in community palliative care services--a literature review | Wrong exposure |
| Rawlings, Deb and Devery, Kim | 2015 | Near death experience and nursing practice: lessons from the palliative care literature | Wrong exposure |
| Raymond, M. and Warner, A. and Davies, N. and Nicholas, N. and Manthorpe, J. and Iliffe, S. | 2014 | Palliative and end of life care for people with dementia: lessons for clinical commissioners | Wrong exposure |
| Robinson, L. and Poole, M. and McLellan, E. and Lee, R. and Amador, S. and Bhattarai, N. and Bryant, A. and Coe, D. and Corbett, A. and Exley, C. and Goodman, C. and Gotts, Z. and Harrison-Dening, K. and Hill, S. and Howel, D. and Hrisos, S. and Hughes, J. and Kernohan, A. and Macdonald, A. and Mason, H. and Massey, C. and Neves, S. and Paes, P. and Rennie, K. and Rice, S. and Robinson, T. and Sampson, E. and Tucker, S. and Tzelis, D. and Vale, L. and Bamford, C. | 2020 | Supporting good quality, community-based end-of-life care for people living with dementia: the SEED research programme including feasibility RCT | Wrong study type |
| Rolls, Kaye Denise and Hansen, Margaret Mary and Jackson, Debra and Elliott, Doug | 2019 | Why Health Care Professionals Belong to an Intensive Care Virtual Community: Qualitative Study | Wrong exposure |
| Ruiz-Iniguez, R. and Bravo Sobrino, N. and Pena Ibanez, F. and Navarro Siguero, N. and Seco Martinez, A. and Carralero Montero, A. | 2017 | Nursing intervention on quality of life in people with home-based palliative care: a systematic review | Wrong language |
| Sacks, Jodi L. | 2013 | Suffering at End of Life | Wrong exposure |
| Sampaio FlorÃªncio, Raquel and Ribeiro Feitosa Cestari, Virna and Campos de Souza, Lorena and Caboclo Flor, Amanda and Pessoa Nogueira, VitÃ³ria and MagalhÃ£es Moreira, Thereza Maria and de GÃ³es Salvetti, Marina and Mendes de Paula Pessoa, Vera LÃºcia | 2020 | Palliative care amidst the COVID-19 pandemic: challenges and contributions | Wrong exposure |
| Saunders S, Killackey T, Kurahashi A, Walsh C, Wentlandt K, Lovrics E, Scott M, Mahtani R, Bernstein M, Howard M, Tanuseputro P, Goldman R, Zimmermann C, Aslakson RA, Isenberg SR | 2020 | Erratum to Palliative Care Transitions From Acute Care to Community-Based Care-A Systematic Review [Journal of Pain and Symptom Management 58 (2019) 721-734.e1] | Wrong study type |
| Saunders, S. and Killackey, T. and Kurahashi, A. and Walsh, C. and Wentlandt, K. and Lovrics, E. and Scott, M. and Mahtani, R. and Bernstein, M. and Howard, M. and Tanuseputro, P. and Goldman, R. and Zimmermann, C. and Aslakson, R. A. and Isenberg, S. R. and American Academy of, Hospice and Palliative Medicine Research Committee Writing, Group | 2019 | Palliative Care Transitions From Acute Care to Community-Based Care-A Systematic Review | Wrong exposure |
| Sawatzky, Richard and Porterfield, Pat and Roberts, Della and Lee, Joyce and Liang, Leah and Reimer-Kirkham, Sheryl and Pesut, Barb and Schalkwyk, Tilly and Stajduhar, Kelli and Tayler, Carolyn and Baumbusch, Jennifer and Thorne, Sally | 2017 | Embedding a Palliative Approach in Nursing Care Delivery: An Integrated Knowledge Synthesis | Wrong exposure |
| Scheerens, C. and Chambaere, K. and Pardon, K. and Derom, E. and Van Belle, S. and Joos, G. and Pype, P. and Deliens, L. | 2018 | Development of a complex intervention for early integration of palliative home care into standard care for end-stage COPD patients: A Phase 0-I study | Wrong study type |
| Schill, Kaela and Caxaj, Susana | 2019 | Cultural safety strategies for rural Indigenous palliative care: a scoping review | Wrong population |
| Scott, M. and Shaver, N. and Lapenskie, J. and Isenberg, S. R. and Saunders, S. and Hsu, A. T. and Tanuseputro, P. | 2020 | Does inpatient palliative care consultation impact outcomes following hospital discharge? A narrative systematic review | Wrong exposure |
| Sebastian, S. A., Shah, Y., Arsene, C. | 2024 | Effectiveness of integrated palliative care telehealth intervention in patients with chronic heart failure: A systematic review and meta-analysis of randomized controlled trials | Wrong exposure |
| Seijas, V., Maritz, R., Mishra, S., Bernard, R. M., Fernandes, P., Lorenz, V., Machado, B., Posada, A. M., Lugo-Agudelo, L. H., Bickenbach, J., Sabariego, C. | 2024 | Rehabilitation in primary care for an ageing population: a secondary analysis from a scoping review of rehabilitation delivery models | Wrong exposure |
| Seow, H. and Bainbridge, D. | 2018 | A Review of the Essential Components of Quality Palliative Care in the Home | Wrong study type |
| Sheehan, O. C. and Ritchie, C. S. and Fathi, R. and Garrigues, S. K. and Saliba, D. and Leff, B. | 2016 | Development of Quality Indicators to Address Abuse and Neglect in Home-Based Primary Care and Palliative Care | Wrong exposure |
| Smith, C. and Newbury, G. | 2019 | Palliative care for community patients diagnosed with dementia: a systematic review | Wrong population |
| Smith, C. and Riley, J. and Owens, M. and Dodds, N. | 2010 | Can we bridge the gap? Hospital2Home: the unity of acute and community end of life care | Wrong study type |
| Smith, S. and Brick, A. and O'Hara, S. and Normand, C. | 2014 | Evidence on the cost and cost-effectiveness of palliative care: a literature review | Wrong exposure |
| Smyth, J. A. and Dempster, M. and Warwick, I. and Wilkinson, P. and McCorry, N. K. | 2018 | A Systematic Review of the Patient- and Carer-Related Factors Affecting the Experience of Pain for Advanced Cancer Patients Cared for at Home | Wrong exposure |
| Söylemez, B. A., Özgül, E., Küçükgüçlü, Ö. & Yenerm G. | 2022 | Telehealth applications used for self-efficacy levels of family caregivers for individuals with dementia: A systematic review and Meta-analysis | Wrong population |
| Speyer, Renée, Denman, Deborah, Wilkes-Gillan, Sarah, Chen, Yu-Wei, Bogaardt, Hans, Kim, Jae-Hyun, Heckathorn, Dani-Ella, Cordier, Reinie | 2018 | Effects of telehealth by allied health professionals and nurses in rural and remote areas: a systematic review and meta-analysis | Wrong population |
| Steers, J. and Brereton, L. and Ingleton, C. | 2007 | Palliative care for all? A review of the evidence in community hospitals | Wrong exposure |
| Steindal, S. A. and Nes, A. A. G. and Godskesen, T. E. and Dihle, A. and Lind, S. and Winger, A. and Klarare, A. | 2020 | Patients' Experiences of Telehealth in Palliative Home Care: Scoping Review | Wrong exposure |
| Steindal, S. A., Nes, A. A. G., Godskesen, T. E., Holmen, H., Winger, A., Osterlind, J., Dihle, A. & Klarare, A. | 2023 | Advantages and Challenges of Using Telehealth for Home-Based Palliative Care: Systematic Mixed Studies Review | Wrong exposure |
| Stone, P. and Kalpakidou, A. and Todd, C. and Griffiths, J. and Keeley, V. and Spencer, K. and Buckle, P. and Finlay, D. A. and Vickerstaff, V. and Omar, R. Z. | 2021 | Prognostic models of survival in patients with advanced incurable cancer: the PiPS2 observational study | Wrong study type |
| Stosz | 2008 | Literature Review of the Evidence Base for a Hospice at Home Service | Wrong exposure |
| Swetz, K. M. and Kamal, A. H. | 2018 | Palliative Care | Wrong exposure |
| Thomas, R. E. and Wilson, D. and Sheps, S. | 2006 | A literature review of randomized controlled trials of the organization of care at the end of life | Wrong exposure |
| Thome, B. and Dykes, A. K. and Hallberg, I. R. | 2003 | Home care with regard to definition, care recipients, content and outcome: systematic literature review | Wrong exposure |
| Totten, A. M. and White-Chu, E. F. and Wasson, N. and Morgan, E. and Kansagara, D. and Davis-O'Reilly, C. and Goodlin, S. | 2016 | Home-Based Primary Care Interventions [Internet] | Wrong exposure |
| Tran, D. and Lai, S. and Salah, R. and Wong, A. and Bryon, J. and McKenna, M. and Chan, Y. | 2020 | Rapid De-Escalation and Triaging Patients in Community-Based Palliative Care | Wrong study type |
| Travis, S. S. and Bernard, M. and Dixon, S. and McAuley, W. J. and Loving, G. and McClanahan, L. | 2002 | Obstacles to palliation and end-of-life care in a long-term care facility | Wrong study type |
| Tsui, E. K. and Wang, W. Q. and Franzosa, E. and Gonzalez, T. and Reckrey, J. M. and Sterling, M. R. and Baron, S. | 2020 | Training to Reduce Home Care Aides' Work Stress Associated with Patient Death: A Scoping Review | Wrong population |
| Vaartio-Rajalin, H. and Fagerstrom, L. | 2019 | Professional care at home: Patient-centredness, interprofessionality and effectivity? A scoping review | Wrong exposure |
| Vaismoradi, M. and Behboudi-Gandevani, S. and Lorenzl, S. and Weck, C. and Paal, P. | 2021 | Needs Assessment of Safe Medicines Management for Older People With Cognitive Disorders in Home Care: An Integrative Systematic Review | Wrong exposure |
| van Crevel, H. | 1996 | [From evidence to treatment] | No full text available |
| van der Steen, J. T. and Goodman, C. | 2015 | What research we no longer need in neurodegenerative disease at the end of life: the case of research in dementia | Wrong study type |
| van Vuuren, J. and Thomas, B. and Agarwal, G. and MacDermott, S. and Kinsman, L. and O'Meara, P. and Spelten, E. | 2021 | Reshaping healthcare delivery for elderly patients: the role of community paramedicine; a systematic review | Wrong exposure |
| Vandersman, Priyanka and Perimal-Lewis, Lua and Tieman, Jennifer | 2021 | Is There an App for That? A Scoping Review of Apps for Care Workers in the Aged Care Sector | Wrong exposure |
| Ventura Mde, M. | 2016 | Effectiveness and cost-effectiveness of home palliative care services for adults with advanced illness and their caregivers | Wrong study type |
| Vernon, E. and Hughes, M. C. and Kowalczyk, M. | 2022 | Measuring effectiveness in community-based palliative care programs: A systematic review | Wrong study type |
| Walsh, R. I. and Mitchell, G. and Francis, L. and van Driel, M. L. | 2015 | What Diagnostic Tools Exist for the Early Identification of Palliative Care Patients in General Practice? A systematic review | Wrong exposure |
| Walshe, C. and Todd, C. and Caress, A. and Chew-Graham, C. | 2009 | Patterns of access to community palliative care services: a literature review | Wrong exposure |
| Wegleitner, K. and Schuchter, P. | 2018 | Caring communities as collective learning process: findings and lessons learned from a participatory research project in Austria | Wrong study type |
| Wei, L. and Walters, J. and Guo, Q. and Fetherston, C. and O'Connor, M. | 2019 | Meaningful and culturally appropriate palliative care for Chinese immigrants with a terminal condition: a qualitative systematic review protocol | Wrong exposure |
| Wenger, N. S. and Rosenfeld, K. | 2001 | Quality indicators for end-of-life care in vulnerable elders | Wrong exposure |
| While, A. and Kiek, F. | 2009 | Chronic heart failure: promoting quality of life | Wrong exposure |
| Wilkinson, E. K. and Salisbury, C. and Bosanquet, N. and Franks, P. J. and Kite, S. and Lorentzon, M. and Naysmith, A. | 1999 | Patient and carer preference for, and satisfaction with, specialist models of palliative care: a systematic literature review | Wrong exposure |
| Yadav, S. and Heller, I. W. and Schaefer, N. and Salloum, R. G. and Kittelson, S. M. and Wilkie, D. J. and Huo, J. | 2020 | The health care cost of palliative care for cancer patients: a systematic review | Wrong exposure |

# **Supplemental material 4: Characteristics of Included Reviews**

| **Author & country** | **Type of review** | **Time period of the search** | **Number and type of studies included** | **Population (number)** | **Interventions** | **Primary outcomes** | **Quality appraisal in study (Yes/ No & tool)** | **Study quality (AMSTAR= grading or SANRA= score)** |
| --- | --- | --- | --- | --- | --- | --- | --- | --- |
| Ahn et al., 2020 ^45^ | Systematic review | 2007-2018 | n= 12 | n= 1361 | Caregiver support intervention | Psychological distress, caregiving burden, quality of life, self-efficacy, and competence for caregiving. | Yes | AMSTAR - 2 rating: Critically low |
|  |  |  | RCTs (n=10), Quasi-experimental study (n=2) |  |  |  | Cochrane Risk of Bias (RoB) tool for RCTs and ROBINS-I |  |
| United States of America |  |  |  | Caregivers of patients with advanced cancer (Stage III or IV) |  |  |  |  |
| Aoun et al., 2014^90^ | Narrative review | 2002-2013 | n= 34 | n= not provided | Care aid support & Personal alarm use | Place of care & death & physical and psychological wellbeing. | No | SANRA Score= 11 |
| Australia |  |  | Retrospective (n= 19), Longitudinal (n=3), Qualitative (n= 3), Prospective (n= 8), Cross-sectional (n= 1) | Terminally ill adults who live alone at home, adult caregivers of people who died 9 months earlier, healthcare professionals caring for these patients |  |  |  |  |
| Bainbridge et al., 2016 ^11^ | Review of systematic reviews | 1950-2014 | n= 40 | n= not provided | Multidisciplinary Specialist palliative care in-home services; Multidisciplinary Palliative care hospital outreach teams | Quality of life (QoL), care satisfaction, individual performance status, pain management, supporting home death, non-pain symptom management. | No | AMSTAR - 2 rating: Critically low |
| Canada |  |  | RCT (n= 13),  Quasi- experimental study (n=4),  Cohort (n= 15),  Pre/post (n=8) | Home treatment programs for people with an advanced or palliative illness. |  |  |  |  |
| Basile et al., 2023 ^46^ | Scoping review | NA | n= 14 | n= not provided | Not specified by paper, but under model of telehealth | Process of care, assessment of patient needs, goal setting, care plans, outcome monitoring, intervention reporting frequency, communication effectiveness, and patient perspectives on positive or negative aspects of technology. | No | Adhered to the Preferred Reporting Items for Systematic Reviews and Meta-analyses Extension for Scoping Reviews (PRISMA-ScR) checklist |
|  |  |  | Quantitative (n= 8), Qualitative (n= 2), Mixed methods (n=4) | Adults aged over 65 in receipt of palliative and/or end-of-life care at home and using technology. |  |  |  |  |
| Italy |  |  |  |  |  |  |  |  |
| Bayly et al., 2021 ^47^ | Tertiary systematic review | 2000-2019 | n= 78 | n= 17,739 | Geriatric Integrated Care AND Palliative Integrated Care | QoL, Utilisation of acute services or community services, Costs of health services utilisation, | Yes | AMSTAR - 2 rating: Low |
| United Kingdom |  |  | Review-level literature that can include trials that are randomised (cluster, parallel, single stage or cross-over design), nonrandomised trials, controlled before after studies, interrupted time series studies and repeated measures studies. | Older people aged 60 years and over with advanced progressive health conditions. |  |  | A Measurement Tool to Assess systematic Reviews (AMSTAR) |  |
| Brereton et al., 2017 ^10^ | Review of systematic & narrative reviews | 2000-2014 | n= 18 | n= not provided | 1. Home-based palliative care (i.e. models of palliative care delivered within the patient or their carer’s own home); 2. Models delivered across multiple settings. 3. Palliative care approach (including outpatient palliative care. 4. Palliative care teams. | Patient outcomes and family or caregiver outcomes. Patient and caregiver outcomes relate to physical (e.g. physical symptoms), psychological (e.g. coping) and social issues (e.g. place of care) | Yes | AMSTAR - 2 rating: Critically low |
|  |  |  |  | Reviews considering adults (defined as people aged 18 and over) with life-limiting illnesses as defined by the study authors. |  |  | A Measurement Tool to Assess systematic Reviews (AMSTAR) |  |
| United Kingdom |  |  | Systematic Reviews (n=9),  Narrative reviews (n=9) |  |  |  |  |  |
| Carmont et al., 2018 ^91^ | Systematic review | Not stated | n= 17 | n= not provided | Integrated primary and secondary care delivering care at home. | Performance status, pain intensity, Hospitalisation, QoL, symptoms, time to death or readmission, all cause hospital bed days, Ed visits, Ed Admissions that then led to later discharge, Recommendations followed in care plan, Perceived usefulness of case, Opinions of community healthcare providers regarding palliative care service, Qualitative analysis of GP lead case conference. | No | AMSTAR - 2 rating: Low |
| Australia |  |  | RCT (n=3),  Cohort (n=1),  Surveys (n=4),  Qualitative (n=7),  Narrative (n=2) | Adults receiving care from their GP, specialist hospital services or an integrated model of care. |  |  |  |  |
| Chen et al., 2022 ^48^ | Systematic review | Up to 2020 | n= 5 | n= 3936 | Home health care in comparison to alternative palliative care | Advance care planning, place of death, Healthcare utilisation. | Yes | AMSTAR - 2 rating: Critically low |
| United Kingdom, Taiwan, The Netherlands, Belgium |  |  | Prospective cohort study (n=1),  Retrospective cohort study (n=2), Case control studies (n=2) | Adults receiving home health care |  |  | The Critical Appraisal Skills Programme (CASP) toolkit |  |
| Chen et al., 2023 ^49^ | Systematic review | Up to 2021 | n= 30 | n= not provided | Telehealth palliative care interventions | Quality of life, burden, survival, mood, advance care planning (ACP), utilisation, satisfaction | Yes | AMSTAR - 2 rating: Moderate |
| China |  |  | RCT (n=30) | Adult patients (aged 18 and older) with life-limiting illness (defined by classifications of disease severity) |  |  | Cochrane Collaboration’s tool. |  |
| Critchley et al., 1999 ^102^ | Systematic review | From 1966 to 1997 | n= 41 | n= not provided | Hospice care model, home care model, home based palliative care nursing service model, hospice at home model. | Health service outcomes, patient outcomes and family or caregiver outcomes. Patient and caregiver outcomes relate to physical (e.g. physical symptoms), psychological (e.g. coping) and social issues (e.g. place of care). | Yes | AMSTAR - 2 rating: Critically low |
|  |  |  | RCTs (n=11), Non-randomized comparative studies (n=30). | Adults receiving palliative care |  |  | Jadad Scale |  |
| Canada |  |  |  |  |  |  |  |  |
| Davies and Higginson., 2005 ^98^ | Systematic review | Up to 2003 | n= 15 | Adults with cancer attending day care facilities | Specialist palliative day care services | Symptom control, quality of life, social and psychological support, and patient and relative satisfaction with care | Yes | AMSTAR - 2 rating: Critically low |
|  |  |  | 15 papers report on data from 12 observational studies. |  |  |  | Author-developed scale |  |
| United Kingdom |  |  |  |  |  |  |  |  |
| Davis et al., 2015 ^103^ | Systematic review | Not provided | n= 37 | n= not provided | Earlier palliative care integration at home and as an outpatient. | Symptoms, QoL, hospitalisations, cost, caregiver quality of life, caregiver burden | No | AMSTAR - 2 rating: Critically low |
| United States of America |  |  | Sixteen RCTs of outpatient palliative care and 13 RCTs of palliative home care, 7 SRs | Patients with a serious illness as an outpatient and at home. |  |  |  |  |
| de Nooijer et al., 2020 ^50^ | Systematic review | Up to 2019 | n= 10 | n= not provided | Specialist palliative care | Symptoms, quality of life, satisfaction with care, hospitalisation, place of death | Yes | AMSTAR - 2 rating: Low |
| Belgium |  |  | Qualitative design (n=3),  Quantitative design (n=3),  Mixed Methods (n=3),  Narrative review (n=1) | Studies concerning older people in the primary/ community care setting using palliative care services |  |  | Qualitative and quantitative assessment scales |  |
| DeGroot et al., 2020 ^51^ | Integrative review | Up to 2019 | n= 19 | n= not provided | A team delivering palliative care to patients with advanced heart failure or supporting their caregivers | Symptom, function status, QoL, symptom burden, advanced care planning, hospitalisation | Yes | AMSTAR - 2 rating: Critically low |
| United States of America |  |  | Quantitative (n=6),  RCT (n=6),  Qualitative (n=6),  Mixed methods (n= 1) | Patients with palliative care receiving out-patient, home based, or community based palliative care, their professionals or caregivers |  |  | Johns Hopkins Nursing Evidence-Based Practice Appraisal tools |  |
| Disalvo et al., 2021 ^52^ | Systematic review of reviews 'meta-review' | 1997- 2019 | n= 16 | n= 31718 (incl. patients/ caregivers/ HCPs) | Digital health technologies | Symptom management/reduction, Patient anxiety/depression/psychological wellbeing, Pain assessment/management, Caregiver quality of life -Caregiver mood/anxiety - Caregiver perception of pain management, Physical function - Caregiver burden, Family functioning, Hospital/ED admissions and/or readmissions, | Yes | AMSTAR - 2 rating: Moderate |
|  |  |  | Integrative review = 2,  Systematic scoping review = 2,  Systematic review = 9, Rapid review of systematic reviews = 1,  Systematic mixed-methods review = 1,  Systematic review of systematic reviews = 1 | People with palliative care needs and/or their carers at home (All ages/no restrictions on age) |  |  | Risk of Bias in Systematic Reviews (ROBIS) phase 2 domains and phase 3 |  |
| Australia |  |  |  |  |  | Patient and/or carer perceptions/user satisfaction/satisfaction with care, experience measures, Cost/cost-effectiveness, Efficacy/effectiveness, Barriers/facilitators of intervention - Features for family support and caregiving functionality – Compatibility, Patient acceptance/compliance of intervention |  |  |
| Ebneter et al., 2022 ^53^ | Scoping review | 2010 onward | n= 27 | n= not provided | Telemedicine intervention | Feasibility, acceptability and needs of patient | Yes | Adhered to the Preferred Reporting Items for Systematic Reviews and Meta-analyses Extension for Scoping Reviews (PRISMA-ScR) checklist |
| Switzerland |  |  | Systematic review (n=3),  RCT (n=1),  Cohort study (n=2),  Cross-sectional survey (n=2),  Non-randomised trial (n=1),  Single-arm intervention study (n=9),  Qualitative study (n= 4),  Review - other (n =3), Other design (n=2) | Adult in/outpatient palliative care patient/caregivers |  |  | Physician Data Query (PDQ®) level of evidence developed by the National Cancer Institute specifically for studies in the field of supportive and palliative care |  |
| Fasolino et al., 2023 ^54^ | Rapid review | January 2020 and January 2023 | n=22 | n = not provided | Telehealth for palliative services and hospice care in rural communities | Assessed outcomes at 3 levels (person- and caregiver-centred, provided-centred, and organizational-centred) | No | AMSTAR - 2 rating: Critically low |
|  |  |  | Clinical Trials (n=5),  Other interventional studies (n=3),  Reviews (n=3),  Surveys (n=2),  Observational/Retrospective (n=2),  Qualitative (n=2), Other (n=5) | Participants in the rural US in rural communities who in receipt of telemedicine for palliative care and hospice services. |  |  |  |  |
| United States of America |  |  |  |  |  |  |  |  |
| Feliciano et al., 2024 ^55^ | Systematic review | 2013-2023 | n=9 | n= 27552 | Home-based Palliative Care | Quality of life (QoL), symptom control/burden, healthcare resource utilization (hospital admissions, emergency department visits (EDV), and length of hospital stay (LHS)) and place of death. | Yes | AMSTAR - 2 rating: Critically Low |
|  |  |  |  | Adults (≥18 years old) of any gender diagnosed with severe, prolonged, or progressive illnesses requiring end-of-life care |  |  | The revised Cochrane Risk of Bias (RoB) tool for RCTs and ROBINS-I assessment tool (cohort-type version) |  |
| Portugal |  |  | RCTs (n=5), Retrospective cohort study (n=3), Retrospective population-based study (n=1) |  |  |  |  |  |
| Finlay et al., 2002 ^104^ | Systematic review | Not provided | n=46  Study designs not reported | n= 43 | Palliative Care Team | Other symptoms, Quality of life Satisfaction, Referral to other services Therapeutic, interventions, Carer: satisfaction Burden/morbidity, Carer Satisfaction, death rates, Health service use and costs | No | AMSTAR - 2 rating: Critically low |
|  |  |  |  |  |  |  |  |  |
| United Kingdom |  |  |  | Palliative patients |  |  |  |  |
|  |  |  |  |  |  |  |  |  |
| Finucane et al., 2021 ^56^ | Systematic review of published systematic reviews (i.e. a meta-review) | 2006 - 2018 | n=21 | n = not provided | Digital health interventions (DHI) | No restrictions were placed on outcomes as potentially influenced by palliative care DHIs. | Yes | AMSTAR - 2 rating: Moderate |
|  |  |  | Systematic reviews (n=21) | Terminally ill patients and their families. |  |  | AMSTAR - 2 and Physician Data Query (PDQ) level of evidence |  |
| United Kingdom |  |  |  |  |  |  |  |  |
| Firth et al., 2023 ^27^ | Mixed method systematic narrative review | 1st January 1990 to 1st August 2022 | n= 64 | n= not provided | Not specified - 24-hour access to specialist palliative care; non-specialist palliative care. | Preferred place of death, physical and mental functioning, Emergency department use and economic evaluation | Yes | SANRA Score= 12 |
| United Kingdom, Taiwan |  |  | Quantitative studies (n=38) (6 RCT’s, 5 controlled cohort, 24 observational and 3 pilot studies),  Qualitative studies (n=14),  Mixed methods (n=4),  Service development papers (n=8) | Adults (over 18 years) with advanced illness from malignant or non-malignant disease in the last year of life or their family caregivers receiving out-of-hours palliative care intervention or service. |  |  | **For qualitative/ quantitative or both**: Quality Assessment Criteria for Evaluating Primary Research Papers (QualSyst) **For quality improvement studies:** Quality Improvement Minimum Quality Criteria Set (QI-MQCS) |  |
| Fulton et al., 2019 ^57^ | Systematic review and meta-analysis | Inception to November 2016 | n= 10 | n= 2385 | Integrated palliative outpatient care | QoL, survival, healthcare utilisation, symptom burden, psychological burden, end of life care outcomes, caregiver outcomes | Yes | AMSTAR - 2 rating: Low |
| United States of America |  |  | 8 RCTs and 2 cluster RCTs | Advanced cancer patients using integrated palliative and oncology service and their carers |  |  | Cochrane risk of bias tool for randomized controlled trials (RCTs) and the revised Newcastle—Ottawa Scale for cohort studies. Individual studies were assigned a summary risk of bias score (low, moderate, or high) |  |
| Gomes et al., 2013 ^13^ | Systematic review | Inception to November 2012 | n= 78 | n= 37561 | Home based palliative care | Symptoms, quality of life, advanced care planning, death at home, satisfaction, hospital cost | Yes | AMSTAR - 2 rating: High |
|  |  |  |  |  |  |  | Cochrane Effective Practice and Organisation of Care (EPOC) Review Group for RCTs/CCTs, CBAs and ITSs checklist |  |
| United Kingdom |  |  | RCTs (n=16), Controlled Clinical Trials (CCTs) (n=4), Controlled Before-After Studies (CBAs) (n=2),Interrupted Time Series (ITS) with nested CBA (n=1). | Patients receiving palliative care at home |  |  |  |  |
| Gonzalez-Jaramillo et al., 2021 ^58^ | Systematic review | 2013 to 11 February 2019 | n= 21 | n= 92000 | Home based palliative care | Hospital use, healthcare cost, place of death | Yes | AMSTAR - 2 rating: Low |
| United Kingdom |  |  | Retrospective cohort (n=13),  Quasi-experimental (n=5), RCTs (n=3) | Adults aged greater than 18, at the end of life, with a severe illness or a disease end-stage. |  |  | Joanna Briggs Institute Critical Appraisal Tools Checklists for use in systematic reviews. |  |
| Goodrich et al., 2024 ^59^ | Additional analysis of systematic review evidence | 1st January, 1990 to 1st August 2022 | n=31 | n = not provided | Out-of-hours services | Service utilisation of the community-based palliative care services out-of-hours. This includes: Time of contact, Who contacted, Type of contact, and background of staff delivering the out-of-hours care. | Yes | SANRA Score= 12 |
|  |  |  | Retrospective review (n=12),  Service evaluation (n=11),  Prospective non-randomised study (n=2),  Practice development report (n= 2),  Qualitative study (n=2),  Quasi experimental (n=1), RCT (n=1). | Palliative care patients and their families living in the community setting for example, their own homes |  |  | **For qualitative/ quantitative or both**: Quality Assessment Criteria for Evaluating Primary Research Papers (QualSyst) **For quality improvement studies:** Quality Improvement Minimum Quality Criteria Set (QI-MQCS) |  |
| United Kingdom |  |  |  |  |  |  |  |  |
| Gordon et al., 2022 ^60^ | Rapid review | 2004-2019 | n= 18 | Patients n= 3,213 Healthcare providers n= 250 | Telehealth interventions | Symptom, satisfaction, quality of life | Yes | AMSTAR - 2 rating: Critically low |
| United States of America |  |  | RCT (n=1), Explorative (n=1), Mixed methods (n=7), Descriptive (n=6), Retrospective chart study (n=1), Clinical trial (n=2). |  |  |  | Clinical Practice Guidelines for Quality Palliative Care developed by the National Coalition for Hospice and Palliative Care. |  |
| Hancock et al., 2019 ^61^ | Systematic review | After 2010 | n= 30 | n= not provided | Telehealth initiative in the delivery of palliative care in the UK. | Patient and caregiver satisfaction, number of acute hospital admission, length admission. | Yes | AMSTAR - 2 rating: Critically low |
| United Kingdom |  |  | Qualitative and Mixed methods (n=10),  Clinical Trials (n=4),  Protocols (n=3),  Interventional (not clearly defined (n=4), Observational (n=5), Service evaluations (n=4) | Palliative care patients receiving telehealth interventions. |  |  | Criteria adapted from Wallace et al.’s 2004 paper on meeting the challenge of developing systematic |  |
| Hayes Bauer et al., 2024 ^62^ | Systematic integrative review | January 2011 - February 2023 | n=46 | n = not provided | Telepalliative care | Patient and family perspective and effectiveness of the Telepalliative care model. | Yes | AMSTAR - 2 rating: Critically Low |
|  |  |  | Qualitative (n=19), Quantitative (n=5), and Mixed methods (n=20) | Adult patients 18+ years and their family, engaged in Telepalliative care including generalist and specialist palliative care |  |  | Mixed Methods Approval Tool (MMAT) version 2018 |  |
| Denmark, Australia, Germany |  |  |  |  |  |  |  |  |
| Head et al., 2017 ^93^ | Systematic review | 2006-2016 | n= 11 | n= not provided | An intervention delivered to patients with end of life or palliative care, using telemedicine or telehealth intervention | Symptoms, quality of life, psychological distress, hospitalisation and cost, patient satisfaction | Yes | AMSTAR - 2 rating: Critically low |
| United States of America |  |  | Case report (n=4), Quantitative (n=3), Mixed methods (n=2), Surveys (n=2) | Patients receiving palliative or end -of-life care for a serious condition (i.e. advanced disease, end-stage disease) |  |  | Using the Cochrane Collaboration’s tool for assessing risk of bias |  |
| Hofmeister et al., 2018 ^94^ | Scoping review | After 2000 | n= 53 | n= not provided | Home based palliative care | Resource use, symptom burden, quality of life, satisfaction, caregiver distress, or place of death as the primary outcome Resource use, symptom burden, quality of life, satisfaction, caregiver distress, place of death, cost analysis, or described experiences. | Yes | AMSTAR - 2 rating: Low   Study was conducted using the PRISMA 2009 statement |
| Canada |  |  | Qualitative (n=10), Cohort studies (n=14), RCTs (n=7), Case-control (n=1), Cross-sectional (n=4), Cost analysis (n=7), Quasi-experimental (n=4), Pre-post design (n=6) | Terminally ill patients at the end of life being care for in the home. |  |  | Critical Appraisal Skills Programme Qualitative Checklist |  |
| Hughes et al., 2023 ^63^ | Systematic review | Inception to August 2021 | n=57 | n=48 (Qualitative study only) | Community-based palliative care | Effectiveness of the interventions using the following outcomes from the included studies: Advance care planning, Costs, Death location, Hospice utilisation, Hospital utilisation, Knowledge, Quality of life, Symptoms | Yes | AMSTAR - 2 rating: Critically Low |
|  |  |  | Quantitative (n=25), Qualitative (n=21), and Mixed method (n=11) research articles | Key and vulnerable population such as Rural, Lower-income, Marginalised racial or ethnic groups |  |  | Mixed Methods Approval Tool (MMAT) version 2018 |  |
| United States of America |  |  |  |  |  |  |  |  |
| Janke et al., 2024 ^64^ | Systematic review | inception to 26th January 2023. | n=7 | n= 805 | Palliative care for non-cancer patient groups | Health care costs such as hospital costs, costs borne by patients, or costs borne by patients’ family members | Yes | AMSTAR - 2 rating: Critically Low |
|  |  |  | RCTs (n=7) | Adult patients (≥ 18 years) with non-cancer life limiting illnesses from the following non-cancer disease groups used in the Global Atlas of Palliative Care: lung, heart, cerebrovascular, central nervous system, liver, renal failure, HIV and dementia |  |  | Drummond's checklist |  |
| United Kingdom, India |  |  |  |  |  |  |  |  |
| Johansson et al., 2024 ^65^ | A rapid systematic review | Inception to February 9, 2023 | n=21 | n=22517 (Participants/data source) | Out-of-hours palliative care telephone advice lines | Patient/carer outcomes (health status, wellbeing and coping), and health care system outcomes (impact on the use of care services).  Cost-effectiveness was differentiated as a separate outcome while patient satisfaction and experience were incorporated as facets of patients/carer outcome. | Yes | AMSTAR - 2 rating: Low |
|  |  |  | Quantitative (n=8), Mixed methods (n=5), Qualitative (n=1), Others (n=7) | Adults (i.e. aged ≥18 years) living at home with palliative care needs and family/unpaid carers |  |  | Mixed Methods Approval Tool (MMAT) version 2018 |  |
| United Kingdom |  |  |  |  |  |  |  |  |
| Johnson et al., 2024 ^66^ | Systematic review with meta-analysis and meta-regression | January 1, 2000 to December 28, 2023 | n = 39 | n=6089 | Specialist palliative care (SPC) | Quality of life, and Emotional wellbeing | Yes | AMSTAR - 2 rating: High |
|  |  |  | RCTs (n=39) | Adults (18+) with advanced illness with palliative care needs. |  |  | Cochrane Risk of Bias 2 tool |  |
| United Kingdom, Switzerland |  |  |  |  |  |  |  |  |
| Johnston et al., 2020 ^16^ | Systematic review | 2000 - 2019 | n= 0 | n= 0 | Home based palliative care | Quality of life, survival, cost | Yes | AMSTAR - 2 rating: Low |
| Ireland |  |  | The review found no studies evaluating the effectiveness or cost-effectiveness of out-of-hours palliative care. | Adults (>18 years old) in the last year of life/ with a terminal illness /had other serious medical need, or are carer for someone with these needs |  |  | Critical Appraisal Skills Programme (CASP) |  |
| Kidd et al., 2010 ^100^ | Narrative review | 1999-2009 | n= 20 | n= not provided | Adults or children with palliative care needs, their relatives and carers, or health professionals using telehealth, telemedicine or Information technology within the UK | Three main outcomes of interest were reported  1) Who is using telehealth 2) what is telehealth being used for 3) is telehealth use increasing | No | SANRA Score= 9 |
| United Kingdom |  |  | Descriptive (n=10),  Pilot studies (n=3),  Mixed methods (n=3),  Cohort (n=1),  Qualitative (n=1),  Case study (n=1),  Other (web-based) (n=1) | Adults or children with palliative care needs, their relatives and carers, or health professionals using telehealth |  |  |  |  |
| Kirtania et al., 2023 ^67^ | Rapid review | Inception to June 2022 | n=7 | n = not provided | Home-based Palliative Care | For the Indian context: a) most effective and essential elements necessary for a home-based palliative care; b) challenges and experiences faced by patients/caregivers and healthcare providers; c) important and evident strategies for implementing a home-based palliative care intervention | No | AMSTAR - 2 rating: Critically Low |
|  |  |  | Mixed method systematic review (n=1), Qualitative (n=3), Descriptive (n=1), Integrative review (n=1), Retrospective (n=1) | Patient’s and caregiver’s receiving palliative care at home |  |  |  |  |
| India |  |  |  |  |  |  |  |  |
| Layne et al., 2024 ^68^ | Integrative review | Inception to May 2023 | n=18 | n=9237 | Care coordination | Service delivery, leadership and governance, workforce, financing, technologies and medical products, information and research reviewed at the micro, meso, and macro levels | Yes | AMSTAR - 2 rating: Critically Low |
|  |  |  | RCT (n=4), Qualitative (n=2), Mixed methods (n=4), Cohort (n=2), Case report (n=1), Program descriptions (n=3), and Study protocols (n=2) | Community-dwelling persons living with Alzheimer’s Disease and Related Dementias (ADRDs) and their caregivers. Informal caregivers were defined as spouses, parents, relatives, or friends. |  |  | Mixed Methods Appraisal Tool (MMAT) Version 2018 |  |
| United States of America |  |  |  |  |  |  |  |  |
| Luckett et al., 2013 ^15^ | Systematic review and meta-analysis | From 2011 | n= 32 | n= not provided | Specialist palliative care provided at home | Symptoms, QoL, place of death, cost | Yes | AMSTAR - 2 rating: Critically low |
| Australia |  |  | Systematic reviews (n=23), RCTs (n=9) | People with life limiting illnesses being nursed exclusively in the home environment. Chronic illness was not included in the definition of life limiting illnesses |  |  | Study quality was independently rated using Cochrane grades |  |
| Lupati et al., 2023 ^69^ | Systematic review | January 2012 - September 2019. | n=34 |  | Community based specialist palliative care interventions | 1. Patient-related outcomes, for example, quality-of-life measures, symptom intensity, and survival. 2. Caregiver-related outcomes, for example, caregivers’ burden score. 3. Equity measures—outcomes specific to disadvantaged or Indigenous patient groups, for example, cultural appropriateness of services, uptake of services by disadvantaged or Indigenous groups. 4. Integration of specialist with non-specialist palliative care services, for example, uptake of primary care services in delivering aspects of palliative care such as end-of-life care.  5. Utilization of hospital services, for example, hospitalization, visits to emergency department (ED). | Yes | AMSTAR - 2 rating: Low |
|  |  |  | Observational studies (n=24), RCTs (n=5), Qualitative studies (n=5) | Patients, carers receiving care, or non-specialist palliative care providers (e.g., primary care) accessing support services from community-based specialist palliative care providers. Studies of Indigenous populations were considered part of the main analysis and synthesis |  |  | AMSTAR checklist, Cochrane tool for assessing risk of bias (RoB 2), ROBINS-I and CASP checklist |  |
| New Zealand |  |  |  |  |  |  |  |  |
| Luta et al., 2021 ^70^ | Narrative review of reviews | January 2000 to September 24th 2019 | n= 46 | n= not provided | Any interventions in palliative care for palliative care groups. | Economic outcomes including cost | Yes | SANRA Score= 12 |
| Switzerland and United Kingdom |  |  | Systematic reviews (n=37),  Narrative/Other reviews (n=6),  Meta-analysis/Meta-review (n=3) | Reviews considering terminally ill adults (18 years old and over) and considering patients with varying illnesses, in receipt of palliative interventions |  |  | Using the AMSTAR - 2 tool |  |
| Marshall et al., 2023 ^71^ | Systematic review | January 1, 1990 to May 2022 | n=15 | n=1831 | All novel model of care interventions | Outcomes reported using empirical data. These include: rate of death at patient preferred place, improved care, rate of hospital use, quality of life and mood, increased Kaplan-Meier 1-year survival rates, reduced hospital usage/admissions/length of stay, Symptom, hospital resource usage, functional decline, healthcare personnel and general practitioner visits. | Yes | AMSTAR - 2 rating: Low |
|  |  |  | RCTs (n=2), Mixed method (n=2), Quasi-experimental (n=2), Longitudinal (n=2), Cohort (n=1), Audit (n=1), Cross sectional (n=1), Case series with pre-post-test (n=2), Comparative (n=1) Pseudorandomised control study (n=1) | Children and adults receiving palliative/end of life care; all health care personnel and non-health care personnel who contribute to the delivery of palliative/end of life care; whom are in a rural setting of a high-income country |  |  | Critical Appraisal Skills Programme (CASP) checklists |  |
| Australia |  |  |  |  |  |  |  |  |
| Mathews et al., 2023 ^72^ | Scoping review | inception to June 2020 | n=23 | n=4016 | Telehealth | Patient or caregiver outcomes. These include: physical and psychological symptoms, quality of life, and acceptability or satisfaction, survival, usage patterns, feasibility, healthcare utilization and cost. | Yes | Adhered to the Preferred Reporting Items for Systematic Reviews and Meta-analyses Extension for Scoping Reviews (PRISMA-ScR) checklist |
|  |  |  | RCTs (n=7), Feasibility trials (n=5), Retrospective chart reviews (n=3), Mixed methods (n=4), Qualitative (n=4) | Adults aged over 18 with advanced cancer receiving telehealth interventions |  |  | Template for Intervention Description and Replication (TIDieR) checklist |  |
| Canada |  |  |  |  |  |  |  |  |
| Miranda et al., 2019 ^73^ | Systematic review | Up to 2018 | n= 9 | n= not provided | Specialist palliative care/Non-specialist palliative care | Symptom, Functional status, behavioural symptoms, Place of death, satisfaction | Yes | AMSTAR - 2 rating: Low |
| Belgium and United Kingdom |  |  | Retrospective case-control (n=2); retrospective cross-sectional (n=1); RCT (n=5); and an unclear design (n=1) | Patients with dementia, living at home, and receiving palliative care at home. |  |  | Quality Assessment Tool for Quantitative Studies’ developed by Effective Public Health Practice Project |  |
| Mojtahedi and Shen, 2023 ^74^ | Scoping review | Not provided | n= 12 | n= 21014 | A palliative care team delivering home hospice care through the pandemic | Perspectives of patients/caregivers/healthcare providers on home palliative care during the COVID-19 pandemic | No | No adherence to PRISMA-ScR or equivalent |
| United States of America |  |  | Qualitative (n=10), Quantitative (n=2) | Patients and caregivers receiving home palliative care or healthcare providers delivering palliative care during the COVID 19 pandemic. Home care was defined as being received at home either face to face or through telehealth. |  |  |  |  |
| Nordly et al., 2016 ^90^ | Systematic review | 2000-2015 | n= 8 | n= not provided | Specialist Palliative care delivered primarily in the home by a team with a least a doctor or nurse on the team | Place of death, survival, quality of life, performance status, pain and dyspnoea | Yes | AMSTAR - 2 rating: Critically low |
| Denmark |  |  | Longitudinal Observational (n=2); Observational cross sectional (n=4); Interventional before and after (n=2) | Patients receiving home-based specialist palliative care with advanced cancer. Advanced cancer defined and incurable or metastatic |  |  | Not stated |  |
| O’Connor et al., 2022 ^75^ | Scoping review | 1995 -2020 | n= 18 | n= not provided | Community based palliative care services for people with dementia and their carers | Caregivers: Pain, QoL, satisfaction, ACP, caregiver burden, place of death, functional status, behavioural symptoms, burden, Depression, functional and cognitive status, hospital admissions, number of referrals to hospice palliative care team | Yes | No adherence to PRISMA-ScR; Utilised Levac et al., (2010) methodology for Scoping reviews |
| Ireland |  |  | Randomised control trials (RCTs) (n =12), Cohort (n=3), Other observational designs (n=3) | People with moderate to severe dementia of any type, or carers of people with dementia, living in the community in their family homes, or a nursing home setting. |  |  | Guidance by Scoping studies: advancing the methodology. Implementation science |  |
| Patton et al., 2021 ^76^ | Systematic review | 2019-2021 | n= 10 | n= not provided | Specialist palliative care & outpatient consultations | Pain, Other symptoms, Quality of life, Satisfaction, Referral to other services, Satisfaction, Burden/morbidity, Home death rates, Health service use and costs, Increased adherence to guidelines, Prescribing rationale Health care/voluntary sector costs | Yes | AMSTAR - 2 rating: Critically Low |
| United Kingdom |  |  | Retrospective (n=6), Longitudinal (n=3), Prospective pilot phase II (n=1) | Adult palliative care patients suffering from cancer. |  |  | Evidence-based librarian (EBL) critical appraisal checklist |  |
| Peeler et al., 2023 ^77^ | Scoping review | Up to 2022 | n= 18 | n= 2508 | Community engaged end of life care | Practical needs (physical, education, psychological, spiritual and social support); Personal growth (knowledge, skills, and attitudes about death and dying; personal reflection and confidence); Community capacity (developing community activists, embedding sustainable change) | No | Adhered to the Preferred Reporting Items for Systematic Reviews and Meta-analyses Extension for Scoping Reviews (PRISMA-ScR) checklist |
| United Kingdom |  |  | RCTs (n = 8), Qualitative (n=6), Quasi-experimental (n=2), Pre-post designs (n=2) | Adults aged 18 and over within the last year of life and their carers, with interventions delivered in the community |  |  |  |  |
| Peerboom et al., 2023 ^78^ | Scoping review | Inception to August 20, 2022 | n=9 | n=2280 | End-of-life communication | Perspectives of the nursing staff, family caregiver, and the older person on end-of life communication | No | Adhered to the Preferred Reporting Items for Systematic Reviews and Meta-analyses Extension for Scoping Reviews (PRISMA-ScR) checklist |
|  |  |  | Qualitative (n=6), Quantitative (n=3) | Nursing staff (i.e., care assistants, certified nursing assistants, licensed vocational nurses, registered nurses, clinical nurse specialists, nurse practitioners), or (family caregivers of) older people in the hospital, nursing home or home care setting. |  |  |  |  |
| The Netherlands |  |  |  |  |  |  |  |  |
| Pinto et al., 2024 ^79^ | Umbrella review | Inception to October 11, 2022 | n=15 | n=141,159 | End of life care | Preferences about place of end-of-life care and death of patients with life-threatening illnesses and their families. | Yes | AMSTAR - 2 rating: Moderate |
|  |  |  | Quantitative (two with meta-analysis) (n=6), Qualitative (n=3), and Mixed-methods (one with meta-analysis) (n=6). | Patients diagnosed with life-threatening illnesses and/or their family members (of any age, gender and race/ethnicity). |  |  | JBI Critical Appraisal Checklist for Systematic Reviews and Research Syntheses. |  |
| Portugal, United Kingdom |  |  |  |  |  |  |  |  |
| Rabow et al., 2013 ^96^ | Narrative review | Not provided | n= 8 | n = not provided | Outpatient non-hospice palliative care | Quality of life, symptom burden, spiritual wellbeing, sleep quality, satisfaction with care, survival and resource use | No | SANRA Score= 9 |
| United States of America |  |  | RCTs (n=8) | Patients with advanced illness (including cancer and non-cancer conditions |  |  |  |  |
| Sánchez-Cárdenas et al., 2022 ^80^ | Systematic review | 2010-2022 | n= 14 | n= 5485 | Telemedicine for patients cared for by primary care teams, those located in rural areas or challenges with travelling, | Symptom management, caregiver support, psychosocial support, pharmacological monitoring, patient follow up, health education, QoL | Yes | AMSTAR - 2 rating: Low |
| Columbia |  |  | Observational (n=6), Experimental (n=6), Qualitative (n=2) | Palliative care for advanced cancer patients with difficulties in accessing standard care (i.e. those living rurally) |  |  | The Newcastle-Ottawa Scale and Standards for Reporting Qualitative Research (SRQR) |  |
| Sani et al., 2024 ^81^ | Systematic review | Inception to October 30, 2023 | n=7 | n=376 | Home-based interventions, such as self-rehabilitation (rehabilitation exercises), educational intervention, structured planned home visits, and pulmonary rehabilitation. | Quality of life (QoL), adherence to treatment, fatigue, bimanual and related activities | Yes | AMSTAR - 2 rating: Moderate |
|  |  |  | RCTs (n=4) and Quasi-experimental studies (n=3) | Patients with different terminal illnesses |  |  | Revised Cochrane risk of bias tool for randomized studies (ROB2) |  |
| Nigeria |  |  |  |  |  |  |  |  |
| Santos et al., 2022 ^82^ | Systematic review | 2015-2019 | n= 7 | n= not provided | Not specified beyond palliative care | Caregiver satisfaction and opinions, cost, place of death | Yes | AMSTAR - 2 rating: Critically low |
| Brazil |  |  | Not stated: objectives and results only | Not stated beyond "elderly" |  |  | Tool unreported |  |
| Santos et al., 2023 ^83^ | Systematic review | 2012– November 2022 | n=8 | n=763 | Palliative care interventions | Quality of life, Symptom burden and control, Anxiety and depression, Advanced care planning, Hospitalisations, and Survival. | Yes | AMSTAR - 2 rating: Low |
|  |  |  | RCTs (n=7) and Cluster-controlled trial (n=1) | Individuals diagnosed with chronic non-malignant respiratory diseases |  |  | Revised Cochrane risk of bias tool for randomized studies (ROB2) |  |
| Portugal |  |  |  |  |  |  |  |  |
| Sarmento et al., 2017 ^97^ | Systematic review | 2000 onward | n= 28 | n= 814 | Home-based palliative care | Experiences and components of home palliative care | Yes | AMSTAR - 2 rating: Low |
| United Kingdom, Portugal and the Netherlands |  |  | Interviews (n=20), Case study (n=2), Cross-sectional surveys (n=5), Secondary analysis of datasets (n=1) | Adult patients (aged 18+) with a life-limiting diagnosis and palliative care needs and/or their family caregivers being cared for at home |  |  | A modified version of the CASP (Critical Appraisal Skills Programme) criteria |  |
| Seica Cardoso et al., 2023 ^84^ | Systematic review | inception until October 2022 | n=4 | n=268 | Non-pharmacological interventions | Patients’ quality of life | Yes | AMSTAR - 2 rating: High |
|  |  |  | RCTs (n=4) | Patients with palliative care needs |  |  | Cochrane risk-­of-­bias (RoB) V.2.0 |  |
| Portugal, United Kingdom |  |  |  |  |  |  |  |  |
| Shepperd et al., 2021 ^85^ | Systematic review | Inception to 2020 | n= 4 | n= not provided | Home-based end-of-life care | Place of death, hospital admissions, patient satisfaction, caregiver satisfaction, Cost | Yes | AMSTAR - 2 rating: High |
| United Kingdom |  |  | RCTs (n=4) | People aged 18 years and over who were receiving end-of-life care at home, at the end of life and required terminal care. |  |  | Cochrane 'Risk of bias' criteria |  |
| Spencer et al., 2024 ^86^ | Rapid review | January 2003 to October 2023 | n=58 | n = not provided | Hospital based model of care, Home/community- based model of care, Hospice based model of care, Mixed models of care | Cost-effectiveness, cost of palliative care or end of life care. | Yes | AMSTAR - 2 rating: Critically low |
|  |  |  | Systematic reviews (n=8), Primary studies (n=50) | Children and adults receiving palliative or end of life care |  |  | Joanna Briggs Institute (JBI) economic evaluations checklist |  |
| United Kingdom |  |  |  |  |  |  |  |  |
| Ventura et al., 2013 ^98^ | Systematic review | 1975 to 2012 | n= 15 | n= 728 | No intervention of interest: qualitative review of unmet needs identified in relevant literature | Unmet needs | Yes | AMSTAR - 2 rating: Critically low |
| Australia |  |  | Qualitative interviews (n=10), Quantitative cross-sectional (n=2), Mixed qual/quant interviews (n=2), Quantitative questionnaire (n=1) | Adult palliative care patients and/or their informal caregivers |  |  | The Standard Quality Assessment Criteria for Evaluating Primary Research Papers from a Variety of Fields |  |
| Vernon et al., 2022 ^87^ | Systematic review | Up to 2021 | n= 63 | n= not provided | Community-based palliative care | Place of death, hospitalisation, Emergency Department (ED) visits, QoL, cost | Yes | AMSTAR - 2 rating: Low |
| United States of America |  |  | Qualitative and mixed methods (n=24) including interviews, surveys, and focus groups, Quantitative (n=49) including surveys, cohorts, case series, cross-sectional, and RCT designs | Those in receipt of community-based palliative care regardless of patient characteristics |  |  | Mixed Methods Appraisal Tool version 2018 |  |
| Walshe and Luker, 2010 ^101^ | Realist review | 1990-2009 | n= 48 | Not stated | District nursing, or equivalent, role in home-based palliative care | Value of in-home nursing; resource availability; DN role definition; resource allocation; symptom management; interprofessional communication; patient and informal caregiver satisfaction; patient and caregiver education; and caregiver resources | Yes | AMSTAR - 2 rating: Critically low |
| United Kingdom |  |  | Qualitative (n=48) |  |  |  | Using the guiding principle for quality appraisal in realist reviews (Pawson, 2006) |  |
| Wicaksono et al., 2024 ^88^ | Scoping review | Inception to April 18, 2023 | n=24 | n = not provided | Home based palliative care | Perspectives of the family caregiver | No | Adhered to the Preferred Reporting Items for Systematic Reviews and Meta-analyses Extension for Scoping Reviews (PRISMA-ScR) checklist |
|  |  |  | Qualitative (n=23), Mixed method (n=1) | Family caregivers of people receiving home palliative care |  |  |  |  |
| The Netherlands, Indonesia |  |  |  |  |  |  |  |  |
| Zheng et al., 2016 ^99^ | Systematic review | 2003-2015 | n= 9 | n= not provided | Telehealth interventions for caregivers of patients receiving palliative care. | Quality of life, anxiety, | Yes | AMSTAR - 2 rating: Critically low |
| United States of America |  |  | Randomised trial (n=2),  Non-randomised interventional (n=3), Cohort study (n=1)  Mixed methods(n=3) | Patients receiving palliative or end-of-life care for a serious condition (i.e., advanced disease, end-stage disease). Caregivers were adults >18yr, relationships to patients included spouses/partners, parents, Children, siblings, grandchildren, daughter-in-law. |  |  | Cochrane Collaboration’s tool for assessing risk of bias |  |
| Zimbroff et al., 2021 ^89^ | Systematic review | Up to 2019 | n= 16 | n= not provided | Home based palliative care interventions | Cost, health service utilisation (including inpatient admissions, hospital days, and skilled nursing facility use), quality of care, and patient/caregiver satisfaction with care, community survival, and mortality rate. | Yes | AMSTAR - 2 rating: Critically low |
| United States of America |  |  | Descriptive/ Observational (n=7),  Retrospective/ Chart-based (n=5),  Analytical (n=1),  Case control (n=1), Expert opinion (n=2) | Homebound Medicaid beneficiaries aged 18 and older in receipt of home-based primary care (HBPC) or home-based palliative care (HBPalC) |  |  | Newcastle-Ottawa Scale for quality assessment |  |

# **Supplemental material 5: Detection of Primary Study Overlap in Included Reviews (Snapshot only)**

| **Primary Studies included in the reviews (n = 1196)** | | **Included Reviews (n=66)** | | | | | | |
| --- | --- | --- | --- | --- | --- | --- | --- | --- |
| **Author and Year** | **Title of Primary Article** | **Ahn et al., 2020** | **Aoun et al., 2014** | **Bainbridge et al., 2016** | **Basile et al., 2023** | **Bayly et al., 2021** | **Brereton et al., 2017** | **Carmont et al., 2018** |
| A Loftus, L., et al. (2007) | Implementation of SIGN 44 guidelines for managing cancer pain in a community setting. | - | - | - | - | - | - | - |
| Aabom, B., et al. (2006). | Does persistent involvement by the GP improve palliative care at home for end-stage cancer patients? | - | - | - | - | - | - | - |
| Aabom, B., et al. (2005) | Population-based study of place of death of patients with cancer: implications for GPs. | - | 1 | - | - | - | - | - |
| Abdul Azeez, E. P. and G. Anbu Selvi (2019). | What determines the sustainability of community‐based palliative care operations? Perspectives of the social work professionals. | - | - | - | - | - | - | - |
| Abela, J. C. (2009) | Symptom management by a community palliative care team | - | - | - | - | - | - | - |
| Abernethy, A. P., et al. (2013) | Delivery strategies to optimize resource utilization and performance status for patients with advanced life-limiting illness: results from the “palliative care trial”[ISRCTN 81117481] | - | - | - | - | - | - | 1 |
| Adam, R., et al. (2015) | Utilising out-of-hours primary care for assistance with cancer pain: a semi-structured interview study of patient and caregiver experiences | - | - | - | - | - | - | - |
| Adam, R., et al. (2018) | What are the current challenges of managing cancer pain and could digital technologies help? | - | - | - | - | - | - | - |
| Adam, R., et al. (2014) | Why do patients with cancer access out-of-hours primary care? A retrospective study. | - | - | - | - | - | - | - |

**Please see attached Excel spreadsheet (Supplemental Material 5_Main Data_Detection of Primary Study Overlap in Included Reviews) for complete information.**

# **Supplemental material 6: Data extraction Template**

| **Field** | **Extracted information** |  | **Example and guidance on how to complete** | **Additional notes, comments or actions required** |
| --- | --- | --- | --- | --- |
| Person extracting the data (Initials) |  | | *First name and surname initials (e.g. SP)* |  |
| Date form was completed |  | | *e.g. 01/12/2021* |  |
| **Publication details and review characteristics** | | | | |
| Review title |  | | *Include the full title of the review* |  |
| Author(s) |  | | *Include all review authors. Example format: Bainbridge D, Hsien S & Sussman J* |  |
| Year |  | | *e.g. 2016* |  |
| Study ID |  | | *e.g. Bainbridge et al. (2016) J Am Geriatr Soc* |  |
| Journal |  | | *Insert the name of the journal* |  |
| Volume |  | | *Insert volume number* |  |
| Issue |  | | *Insert issue number, if available* |  |
| Page(s) |  | | *Insert page numbers, if available* |  |
| DOI |  | | https://doi.org/10.1111/jgs.14025 |  |
| Review type |  | | *Describe the type of review (e.g. scoping review, narrative review, systematic review)* |  |
| Country |  | | *List the country or countries of the review authors* |  |
| Primary contact information review |  | | *Insert email address of corresponding review author* |  |
| Review question/aim |  | | *Insert review question/aim* |  |
| Objective (if listed) |  | | *List objectives, if available. Insert new row if needed.* |  |
| Objective (if listed) |  | | *List objectives, if available. Insert new row if needed.* |  |
| Objective (if listed) |  | | *List objectives, if available. Insert new row if needed.* |  |
| Objective (if listed) |  | | *List objectives, if available. Insert new row if needed.* |  |
| Brief summary of population of interest |  | | *Summarise the population of interest and any definitions of population provided. For example: Participants aged 18 years or older in receipt of a home palliative care service, their family caregivers, or both.* |  |
| Any comparison groups? |  | | *Briefly summarise any comparison groups considered in the included studies, if applicable. For example, usual care.* |  |
| Eligibility criteria | **Inclusion** | **Exclusion** | *List or summarise any inclusion and exclusion criteria stated.   Inclusion criteria example: Inclusion criteria were: 1) must assess at least one component of palliative care; 2) must report location of care as “home of patient” irrespective of if care was delivered in an individual home, hospice, or continuing care; 3) report on any outcome; and 4) report primary data. No study designs were excluded.  State if these are not clearly specified.* |  |
|  |  |  |  |  |
| Number of databases searched |  | | *Provide count.* |  |
| Names of databases searched |  | | *List the names of databases searched.* |  |
| Month and year searched from and to |  | | *Insert the dates that the reviews span (e.g. 1950 to November 2012)* |  |
| Date of updated search, if applicable |  | | *Insert date of updated search, if applicable* |  |
| Is a full search strategy provided |  | | *Summarise whether a full search strategy is available and add a screen shot of the search strategy to the 'Search Terms' tab within this document.  For example: Example of full search strategy is present Abbreviated search strategy present Only provided key concepts of search strategy (e.g. home care) No search strategy present* |  |
| Summary of search strategy |  | | *Copy from article or summarise how search strategy was developed. For example: A scoping review was completed [13]. Quality of included studies was assessed to increase the utility of this scoping review and to add the needed quality lens to the literature. In August 2016, the following electronic databases were searched from inception: PubMed, Embase, Cumulative Index to Nursing and Allied Health Literature (CINAHL), Web of Science, Cochrane Library, EconLit, PsycINFO, Centre for Reviews and Dissemination, Database of Abstracts of Reviews of Effects, and National Health Service Economic Evaluation Database. Palliative care in the home experts were contacted to identify additional papers. Search results were limited to those published after the year 2000, to ensure that included studies were representative of modern palliative home care. Only published studies were reviewed thus ethics approval was not required. Grey literature was not included. Only English search results with human subjects were included in abstract review. Preferred reporting items for systematic reviews and meta-analyses (PRISMA) guidelines were followed to ensure methodological best practices [14]. The search strategy consisted of three concepts. First, terms for palliative care such as “palliative care,” “terminally ill,” and “end of life care” were searched. Second, terms for home care such as “home care services/trends,” “health system pathway,” and “component” were searched. Third, terms for outcomes such as “health care quality, access, and evaluation,” “quality,” and “patient satisfaction” were searched. These three concepts were combined using the Boolean operator “and.”* |  |
| Details of searching any other resources |  | | *Insert details of searching for other resources e.g. hand searches or reference lists* |  |
| Summary of analysis |  | | *Copy text summarising approach to analysis* |  |
| Number of included studies |  | | *Provide count of included studies* |  |
| Types of studies included |  | | *Describe the types of studies included (e.g. RCT (n = 1)* |  |
| Countries covered by the review |  | | *Describe the countries included in the review (e.g. UK (n = 10)* |  |
| Overall findings |  | | *Copy and paste the overall findings.* |  |
| Brief summary of intervention of interest *[To start a new line, press Alt+Enter]* |  | | *Description of intervention of interest and any definitions.   Example: A team delivering home palliative care with the presence of the following four elements:  1. Primarily for patients with a severe or advanced disease (malignant or non-malignant), no longer responding to curative/maintenance treatment or symptomatic (or both), or their family caregivers, or both. 2. Aiming to support patients or family caregivers, or both, outside hospital and other institutional settings as far as possible and to enable patients to stay at home. While conducting the review we have also included interventions in which it was clear the majority of service contacts were established while the patients were at home. Services delivered in skilled nursing facilities, day care centres, residential homes or prisons were excluded. 3. Providing either specialist or intermediate palliative/hospice care, as defined in a previous systematic review 4. Providing comprehensive care and aiming at different physical and psychosocial components of palliative care* |  |
| Primary outcome of interest |  | | *Describe the primary outcome of the review (e.g. symptom burden), add new line if needed. If not specified in advance, summarise the outcomes identified from the studies they included. For example, in Hofmeister et al. (2013):  Multiple outcomes described studies in which the objective statement identified a combination of resource use, symptom burden, quality of life, satisfaction, caregiver distress, or place of death as the primary outcome. The most commonly reported outcome was descriptive in nature with the objective of the study being to describe experiences with services offered.* ***ADD NEW ROWS IF NEEDED*** |  |
| Describe primary measures considered to assess outcome, if specified |  | | *Describe the measure used to assess the primary outcome if available, add new line if needed and may need to consult primary included studies. For example, Edmonton Symptom Assessment Scale (ESAS).* |  |
| Secondary outcome of interest |  | | *Describe any secondary outcome, add new line life needed. If not specified in advance, summarise the outcomes identified from the studies they included. For example, pain or utilisation of care.* |  |
| *Describe measure used to assess secondary outcome.* |  | | *Describe the measure used to assess the secondary outcome, add new line if needed. For example, Integrated Palliative care Outcome Scale (IPOS).* |  |
| Secondary outcome of interest |  | | *Describe any secondary outcome, add new line life needed. If not specified in advance, summarise the outcomes identified from the studies they included. For example, pain or utilisation of care.* |  |
| *Describe measure used to assess secondary outcome.* |  | | *Describe the measure used to assess the secondary outcome, add new line if needed. For example, Integrated Palliative care Outcome Scale (IPOS).* |  |
| Secondary outcome of interest |  | | *Describe any secondary outcome, add new line life needed. If not specified in advance, summarise the outcomes identified from the studies they included. For example, pain or utilisation of care.* |  |
| *Describe measure used to assess secondary outcome.* |  | | *Describe the measure used to assess the secondary outcome, add new line if needed. For example, Integrated Palliative care Outcome Scale (IPOS).* |  |
| Secondary outcome of interest |  | | *Describe any secondary outcome, add new line life needed. If not specified in advance, summarise the outcomes identified from the studies they included. For example, pain or utilisation of care.* |  |
| *Describe measures used to assess secondary outcome.* |  | | *Describe the measure used to assess the secondary outcome, add new line if needed. For example, Integrated Palliative care Outcome Scale (IPOS).* |  |
| *Model(s) of care, if identified* |  | | *Describe the model(s) of care, if specified. For this review, a ‘model of care’ has been defined as the way in which health and care services are delivered and provides ‘a descriptive picture of practice’. For example, Firth and colleagues established key criteria to define and allow for comparison between models of specialist palliative care, such as setting of care (e.g. inpatient hospital, inpatient hospice and home-based) or number of disciplines delivering care.* |  |
| Component 1 |  | | *List each of the components of care identified. For example: integrated teamwork, symptom management, holistic care, skilled providers (who are caring and compassionate), timely and responsive care, and patient and family preparedness. EACH NEEDS TO BE ENTERED ON A SEPARATE LINE. Add a new row, if needed.* |  |
| Component 2 |  | | *List each of the components of care identified. For example: integrated teamwork, symptom management, holistic care, skilled providers (who are caring and compassionate), timely and responsive care, and patient and family preparedness. EACH NEEDS TO BE ENTERED ON A SEPARATE LINE. Add a new row, if needed.* |  |
| Component 3 |  | | *List each of the components of care identified. For example: integrated teamwork, symptom management, holistic care, skilled providers (who are caring and compassionate), timely and responsive care, and patient and family preparedness. EACH NEEDS TO BE ENTERED ON A SEPARATE LINE. Add a new row, if needed.* |  |
| Component 4 |  | | *List each of the components of care identified. For example: integrated teamwork, symptom management, holistic care, skilled providers (who are caring and compassionate), timely and responsive care, and patient and family preparedness. EACH NEEDS TO BE ENTERED ON A SEPARATE LINE. Add a new row, if needed.* |  |
| Strengths |  | | *Copy and paste* |  |
| Limitations |  | | *Copy and paste* |  |
| Conclusions |  | | *Copy and paste* |  |
| Implications for practice |  | | *Copy and paste* |  |
| Implications for research |  | | *Copy and paste* |  |
| Do the review authors need to be contacted for further information? |  | | *Please put "Yes" if there is incomplete information that is required and contact authors, update accordingly.* |  |
